# 4-Vinyl Guaiacol: A Key Intermediate for Biobased Polymers

**DOI:** 10.3390/molecules29112507

**Published:** 2024-05-25

**Authors:** Elena Rigo, Cédric Totée, Vincent Ladmiral, Sylvain Caillol, Patrick Lacroix-Desmazes

**Affiliations:** 1 ICGM, University of Montpellier, CNRS, ENSCM, 34293 Montpellier, France; elena.rigo@umontpellier.fr (E.R.); cedric.totee@enscm.fr (C.T.); vincent.ladmiral@enscm.fr (V.L.); sylvain.caillol@enscm.fr (S.C.); 2 Synthomer Speciality Chemicals SAS, 76430 Sandouville, France; 3 Synthomer Ltd., Harlow CM20 2BH, UK

**Keywords:** biobased, guaiacol, polymers, radical polymerization

## Abstract

In order to contribute to the shift from petro-based chemistry to biobased chemistry, necessary to minimize the environmental impacts of the chemical industry, 2-methoxy-4-vinylphenol (4-vinyl guaiacol, 4VG) was used to synthesize a platform of biobased monomers. Thus, nine biobased monomers were successfully prepared. The synthesis procedures were investigated through the green metrics calculations in order to quantify the sustainability of our approaches. Their radical homopolymerization in toluene solution initiated by 2,2′-azobis(2-methylpropionitrile) (AIBN) was studied and the effect of residual 4VG as a radical inhibitor on the kinetics of polymerization was also explored. The new homopolymers were characterized by proton nuclear magnetic resonance (^1^H-NMR) spectroscopy, size exclusion chromatography and thermal analyses (dynamical scanning calorimetry DSC, thermal gravimetric analysis TGA). By varying the length of the alkyl ester or ether group of the 4VG derivatives, homopolymers with *T*_g_ ranging from 117 °C down to 5 °C were obtained. These new biobased monomers could be implemented in radical copolymerization as substitutes to petro-based monomers to decrease the carbon footprint of the resulting copolymers for various applications.

## 1. Introduction

The current trend in polymer synthesis, in line with the public awareness of the environmental impact related to petro-based chemistry (greenhouse gas emissions [1]), is to replace petro-based compounds [2,3,4,5,6] by biobased compounds [7,8,9,10,11,12,13,14,15,16,17]. The final aim is to produce bio-based materials with at least equal physico-chemical properties and performances in applications compared to the current 100% petroleum-based materials or even superior or additional properties opening new markets not accessible yet. With this aim, chemical producers are looking for greener alternatives and two main routes have been explored from biomass: (1) the so-called “drop-in approach”, which consists of the synthesis of identical structures to conventional petro-based monomers (e.g., bio-based styrene or acrylic acid [18,19]), and (2) the synthesis of novel monomer structures. This work was initiated with the objective of contributing to the development of new bio-based monomers using the second approach. 

**Figure 1:**
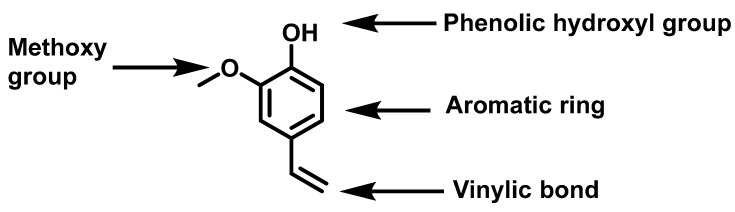
Four reactive sites of 4-vinyl guaiacol (4VG).

Aromatic monomers are widely used in the chemical industry for the properties they confer to the copolymers (high mechanical and thermal stability [20]), but only a few studies report aromatic bio-based monomers able to copolymerize by radical polymerization. Hence, we have synthesized aromatic bio-based monomers derived from lignin. Lignin is a non-edible raw material and the second most abundant renewable polymer after cellulose [21]. Its depolymerization has been extensively studied and a series of potentially promising “platform” molecules have been identified [22]. However, most phenolic molecules derived from lignin do not possess double bonds able to react by chain growth radical polymerization. Hence, other types of polymerization techniques such as step-growth polycondensation have been used, for ferulic acid for example [23,24,25]. Recently, functions such as (meth)acrylates have been introduced into eugenol [26], cardanol [27] and vanillin [9], for example, to prepare monomers suitable for radical polymerization. As those vinylic functions are currently mainly petroleum-based, the addition of these (meth)acrylate groups decreases the bio-based carbons content in the final monomer. Thus, we have chosen 2-methoxy-4-vinylphenol, also known as 4-hydroxy-3-methoxystyrene or 4-vinyl guaiacol (4VG), a phenol already containing a styrenic double bond (amenable to radical (co)polymerization) as platform molecule. 4VG can be easily obtained by condensation of vanillin [28] or by decarboxylation of ferulic acid [29], which makes it a 100% biobased molecule able to react by radical polymerization. It possesses four reactive sites: the methoxy group, the phenol group, the aromatic ring and the vinyl group (Figure 1). Because of the aromatic ring and the vinyl group, the structure of 4VG can be considered as a substitute for styrene, one of the major petro-sourced monomers extensively used in the production of many materials. 

The phenolic hydroxyl group could confer adhesive [30] or anti-viral [31] properties. However, phenols slow down or inhibit radical polymerization, acting as radical scavengers via the formation of hydroquinone intermediates [32]. The direct radical homopolymerization of 4VG has been attempted by Kodaira et al. [33]. However, they observed that the propagating radical reacted by radical transfer onto 4VG and that the produced radical did not propagate, thus leading to the inhibition of the radical polymerization. It was concluded in this study that the spontaneous polymerization of 4VG at room temperature under a nitrogen atmosphere in the dark for 18 months, producing only low molar mass oligomers (*n* = 8), was not due to radical polymerization as in the case of styrene, but was instead caused by cationic polymerization initiated by the acidic hydroxyl group of 4VG. The issue of 4VG being a radical inhibitor caught our attention, in particular because some articles are contradictory. Leisch et al. [34] performed the radical precipitation homopolymerization of 4VG in toluene and they obtained a polymer with a 90% yield, with a *T*_g_ between 86 °C and 92 °C. Leisch et al. also reported the thermal self-polymerization of 4VG in toluene at 100 °C, resulting in 62% monomer conversion (*M*_n_ = 4500 g/mol, *M*_w_/*M*_n_ = 1.5). In contrast, Kamigaito et al. [29] showed that the solution polymerization of 4VG in toluene, by reversible addition–fragmentation chain transfer (RAFT) polymerization, stopped at about 20% monomer conversion. They ascribed the low conversion to the weak inhibition caused by the phenol group of 4VG. To overcome this inhibition effect, the functionalization of the phenol group appears as an efficient method. Following work performed on acetoxy-protected hydroxystyrene [35,36], Kamigaito et al. investigated the reactivity of 4VG derivatives, protected with either acetyl, tert-butyldimethylsilyl or triethylsilyl groups, resulting in their successful radical RAFT polymerization [29]. Similarly, van Schijndel et al. [37] studied the radical polymerization of acetoxy-protected hydroxystyrene derivatives and the properties of the polymers isolated after hydrolysis of acetyl protecting groups. During their work, they suspected that transfer to monomer took place during radical solution polymerization of acetoxy4VG in toluene. Recently, Alexakis et al. [38] prepared some ether-protected 4VG (methyl 2-(2-methoxy-4-vinylphenoxy)acetate, 2-(2-methoxy-4-vinylphenoxy)acetic acid, and 1-(benzyloxy)-2-methoxy-4-vinylbenzene) and showed the ability of these new monomers to polymerize by solution radical polymerization to reach high molar masses (*M*_n_~2.5 × 10^5^ g/mol). Another modification of the phenol group of 4VG was performed via nucleophilic substitution of the chlorine atom of epichlorohydrine to produce an epoxide-derivatized 4VG (4VGEP) [39]. In particular, the authors studied the RAFT homopolymerization of 4VGEP and obtained polymers with low dispersity (1.07) and controlled molar mass (*M*_n_ = 6900 g/mol). 

In summary, very few biobased aromatic monomers have been synthesized so far and the influence of their substituents on the polymer properties has not been studied. In the present work, nine biobased monomers with substituents with different chain lengths were successfully prepared from 4-vinyl guaiacol and characterized for the first time. In order to overcome the 4VG inhibition problem, the phenol group was protected by esterification or alkylation leading to a platform of 4VG-derived monomers using sustainable and industrially amenable approaches. The new biobased aromatic monomers, some of which reaching 100% biobased carbon content, were homopolymerized in toluene under solution conventional radical polymerization conditions. The thermal, chemical and physical properties of the resulting homopolymers were assessed and compared with those of polystyrene. These new, safe and sustainable biobased aromatic monomers and polymers could be instrumental in minimizing some of the environmental impacts of the chemical industry. 

## 2. Results and Discussion

### 2.1. Synthesis of Biobased Monomers Derived from 4-Vinyl Guaiacol

The successful synthesis of a series of nine protected 4VG monomers derived from commercially available bio-based 4-vinyl guaiacol was achieved and their corresponding ^1^H-NMR spectra, as well as their GC-MS and DSC characterizations, are reported in the Supporting Information. 

**Figure 2:**
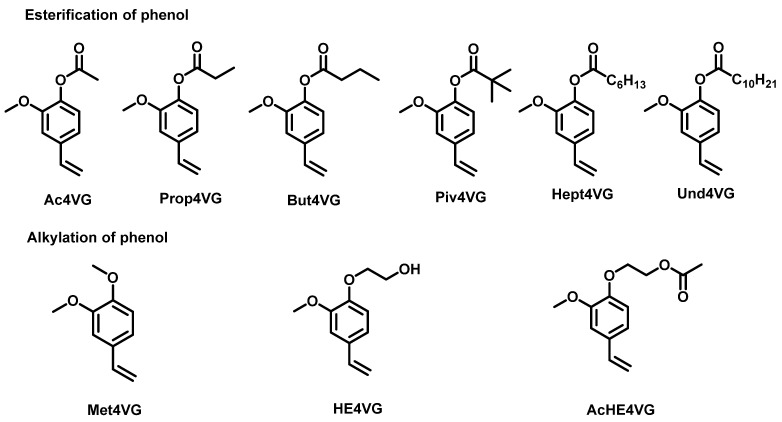
Range of 4VG derivatives synthesized by protection of phenol function.

The protection of phenol function was performed by esterification or alkylation (Figure 2). 

#### 2.1.1. Esterification

**Figure 3:**
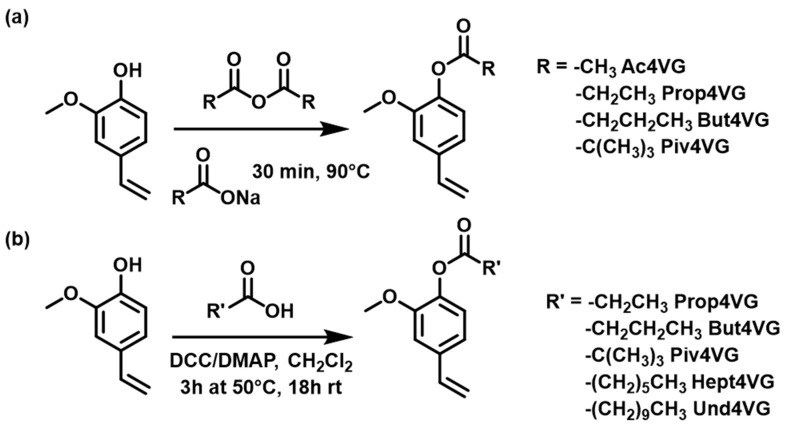
The two synthetic routes used in this work to protect the phenol function by esterification: (**a**) Esterification using the corresponding anhydride; (**b**) Esterification using the corresponding carboxylic acid.

A series of esterified-4VG with C_2_ to C_11_ carboxylic acids was synthesized by esterification of the phenol function. The synthesis of Ac4VG has been reported previously [29,35,36]. In general, we tested two synthesis routes depending on the availability of the starting materials (Figure 3). 

When the required anhydride was commercially available, the protection was performed via a simple reaction with the phenol following the published procedure [37] (Figure 3a). This route afforded a high conversion of 4VG (>92%) with a high level of purity (see the Supporting Information) for all the targeted esters. When the anhydride was not commercially available, we performed Steglich esterification (Figure 3b). This second route produced a series of fully biobased molecules since propionic acid, butyric acid, heptanoic acid and undecanoic acid could be extracted from renewable resources [40,41]. In addition, high gravimetric yields were obtained. However, the conversion of 4VG was slightly lower (90–95%) than those achieved via route 1 and purification by column chromatography was necessary. Moreover, this second strategy (Steglich esterification) was executed using chlorinated solvent (CH_2_Cl_2_) and hard-to-remove additives (DCC and DMAP). These limitations might be mitigated by using, for instance, dimethyl carbonate as a more benign solvent instead of CH_2_Cl_2_, and by selecting easier to remove reagents such as Mukaiyama’s reagent (2-chloro-1-methylpyridinium iodide) [42] or maybe using mecanochemistry processes.

#### 2.1.2. Alkylation

**Figure 4:**
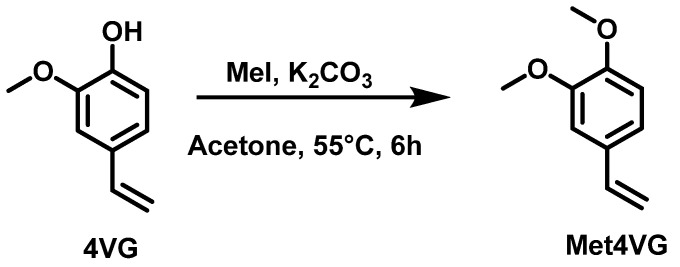
Synthetic strategy used to synthesize Met4VG.

In order to avoid the presence of ester function, which can be prone to hydrolysis, direct methylation using methyl iodide, as a protection strategy of 4VG, was also achieved. The reaction was performed in acetone at 55 °C (reflux) for 6 h. The conversion of 4VG was 96%, and the final monomer was pure and obtained with a good gravimetric yield of 98% (Figure 4).

**Figure 5:**
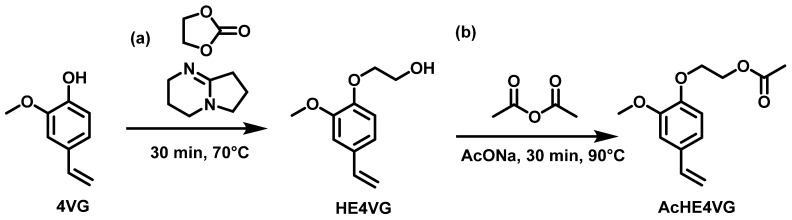
Synthetic strategy used to synthesize AcHE4VG: (**a**) Chain elongation; (**b**) Acetylation.

A chain elongation to introduce an ethyl spacer by alkylation, before esterification, was also performed. The reason for moving the hydroxyl group further from the aromatic ring was to reduce the possibility of hydrolysis of the subsequent ester group (an aliphatic ester is more stable than an aromatic ester) [43] (Figure 5a). The reaction was run in bulk using ethylene carbonate at a high temperature (150 °C) for 30 min using 1,5-diazabicyclo [4.3.0]non-5-ene (DBN) as catalyst following the procedure used on eugenol by Molina Gutièrrez et al. [26] to produce HE4VG. The hydroxyl function of HE4VG was then acetylated by reaction with acetic anhydride (Figure 5b). The synthesis of AcHE4VG was achieved with a low gravimetric yield (35%); however, it can open the way to the synthesis of a range of polymers with interesting properties by (co)polymerization.

Thus, the synthesis of nine 4VG-based monomers was successfully achieved.

#### 2.1.3. Green Metrics

In order to have a first approximation of the sustainability of our synthesis procedures and to enrich the discussion, the green metrics such as atom economy (*AE*), *E-factor*, *C-efficiency*, reaction mass efficiency (*RME*) and the % of renewable carbon atoms (*Biobased carbon*) have been calculated for all the reactions (Table 1). Atom economy measures the conversion efficiency of a chemical process in terms of the atoms actually incorporated into the desired products, assuming a yield of 100% [44]. It is defined as the molar mass of the desired product divided by the sum of the molar masses of all the reactants used in the stoichiometric equation, expressed as a percentage. The higher the *AE*, the more sustainable the reaction, because fewer reactants end up as waste products. *E-factor* measures the amount of waste produced during a process. It is defined as the mass ratio of waste to desired product (expressed in kg of waste per kg of product). It takes into account waste byproducts, leftover reactants, solvent losses and spent catalysts (also supported). Hence, a higher *E-factor* means more waste and higher environmental impact [45]. Carbon efficiency (*C-efficiency*) quantifies the number of carbon atoms in the starting materials that end up in the desired product. It is expressed in % based on the total mass of carbon in the reactants; the higher the *C-efficiency*, the better [46]. Reaction mass efficiency (*RME*) measures the efficiency with which the reactant mass ends up in the desired product [47]. It is defined as the mass of the isolated product divided by the total mass of the reactants and is expressed in %; the higher the *RME*, the better. *Biobased carbon* is the calculation of the amount of renewable carbon in the final monomer; the higher the % of *Biobased carbon*, the better. For esterification reactions, in general, *Route 2* (Steglish esterification starting from the acid) showed a higher atom economy than *Route 1* (esterification using the anhydride), because the latter was performed using a symmetrical anhydride which decreased the final atom economy. However, the *E-factor* values were higher for *Route 2* because dichloromethane (DCM) was employed as a solvent, which meant that the total amount of waste produced during the reaction was higher than for *Route 1*. For the synthesis of Met4VG, the value of atom economy was very low because MeI was used as reactant. The use of a compound with a heavy atom such as iodine (*M* = 126.9 g/mol) is not recommended. The use of other methyl-halides was impossible because their boiling points were too low (*T*_eb(MeCl)_ = −24 °C and *T*_eb(MeBr)_ = 4 °C) and they are less reactive than MeI. The utilization of MeI was the best compromise to easily and quickly obtain Met4VG. The *C-efficiency* and the reaction mass efficiency *RME* were moderate for all the reactions. The *biobased carbon* (%) derived from biomass was elevated and reached 100% in some cases (Prop4VG *route 2*, But4VG *route 2*, Hept4VG, Und4VG) because the corresponding carboxylic acid could be considered as biobased as explained previously. However, these green metrics calculations were performed without taking into account the purification processes which increases the total amount of waste. In addition, the development of new bio-based monomers often does not equate to a more sustainable process because the steps used to extract, purify and synthesize them may be more time-consuming and more energy-intensive than the synthesis of their petroleum-based counterparts.

### 2.2. Solution Homopolymerization of Biobased Monomers Derived from 4-Vinyl Guaiacol

After the synthesis of the novel platform of biobased monomers derived from 4-vinyl guaiacol, the study of their behavior in radical homopolymerization in toluene solution was performed in order to compare their reactivity and study the thermal properties of the new homopolymers.

#### 2.2.1. Homopolymerization of 4-Vinyl Guaiacol and Its Inhibitor Properties

First, we conducted our own investigation of the homopolymerization of 4VG because some articles were contradictory about the phenol capacity to scavenge radicals and inhibit radical polymerization (a hypothetical mechanism of inhibition was attempted in Figure S21), as explained previously.

We carried out the homopolymerization of unprotected 4VG in toluene (at a monomer content of 22 wt%, 1 eq.) at 70 °C, with AIBN as initiator (0.02 g, 0.12 mmol, 0.015 eq.) and 1,4-bis(trimethylsilyl)benzene as internal standard (0.09 g, 0.41 mmol, 0.05 eq.) to monitor the monomer conversion by ^1^H-NMR. After 24 h, the achieved conversion was only 27% and the molar mass was 3700 g/mol (*Đ* = 3.1). This result confirms the observation of Kamigato et al. [29]. The phenol function seems to act as a radical scavenger and inhibits the polymerization. However, through this simple test, we were not capable of quantifying this inhibiting power.

**Figure 6:**
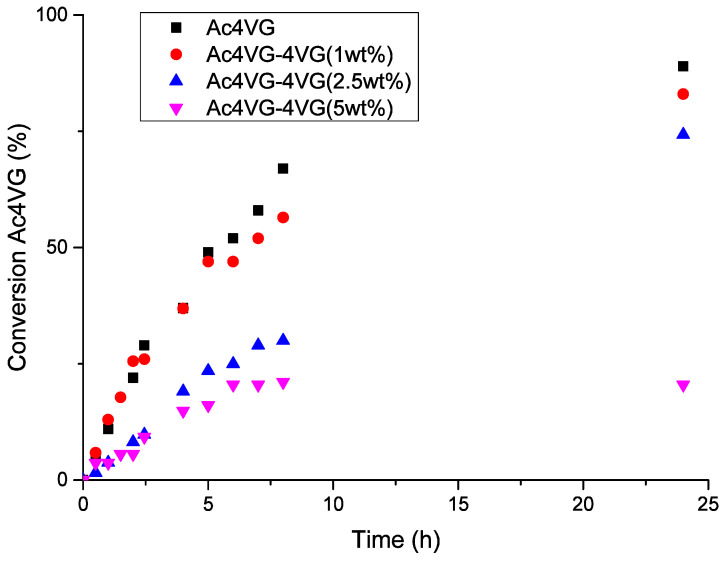
Evolution of Ac4VG conversion versus time, in the presence of increasing amounts of 4VG (0, 1, 2.5, and 5 wt% based on monomers), during radical polymerization. *[Ac4VG]*_0_/*[AIBN]*_0_ = 1/0.05 in toluene at 70 °C.

Additional studies were thus performed to elucidate the effect of 4VG on the radical (co)polymerization of Ac4VG with 4VG (at a constant monomer content of 22 wt%). Known quantities of 4VG were added to the reaction medium during the kinetic monitoring of Ac4VG radical polymerization (Figure 6). The addition of 4VG clearly decreased the conversion of Ac4VG. In particular, when 5 wt% (based on monomers) of 4VG was added, the conversion of Ac4VG only reached 20% in 24 h instead of 89% in the absence of 4VG. This result indicates that residual 4VG in the protected monomers will have an impact on the (co)polymerization rate and on the maximum attainable conversion. Thus, the presence of residual 4VG should be avoided and carefully quantified. In addition, it was observed that molar masses (*M*_n_) (Figure S22*)* decreased only slightly upon increasing the amount of 4VG: *M*_n,Ac4VG(0%wt4VG)_ > *M*_n,Ac4VG(1%4VG)_ > *M*_n,Ac4VG(5%4VG)_ (16,000 g/mol > 14,000 g/mol > 12,500 g/mol, respectively) which could mean that 4VG reacts as a poor chain transfer agent (low re-initiation efficiency after transfer reaction), which is consistent with its inhibitor properties [48].

#### 2.2.2. Homopolymerization of 4-Vinyl Guaiacol Derivatives

The biobased monomers were polymerized via solution polymerization to prepare the corresponding homopolymers and study their thermochemical properties. The solution homopolymerization was performed in toluene (*n*_monomer_ = 7.8 mmol, and the quantity of toluene was adjusted to maintain a constant monomer content of 22 wt%) at 70 °C, with AIBN as initiator and 1,4-bis(trimethylsilyl)benzene as an internal standard to monitor the monomer conversion by ^1^H-NMR (integration of the double bond). For thermal analyses (TGA, DSC), another sample without 1,4-bis(trimethylsilyl)benzene was synthesized to avoid any effect of the internal standard (e.g., plasticizer) on the thermal properties. SEC analyses were performed on the crude sample taken before polymer purification.

**Figure 7:**
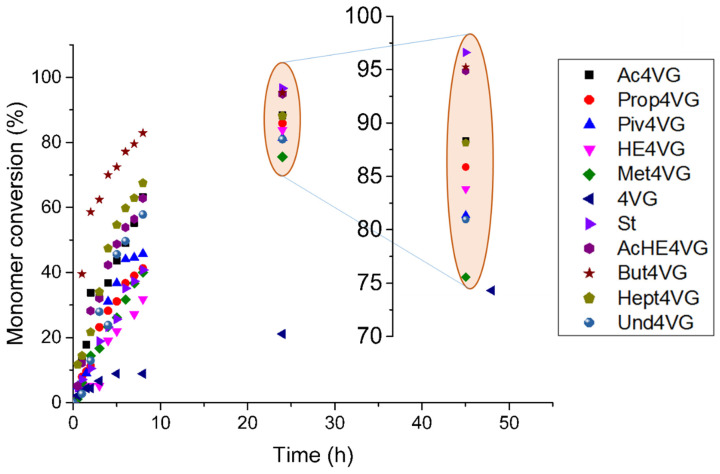
Monomer conversion versus time for the homopolymerization of 4VG derivatives initiated by AIBN at 70 °C in toluene (22 wt% monomer content). The numerical data are reported in Table S2.

Since 4-vinyl guaiacol is a styrene-like biobased monomer, we compared the homopolymerization rates and conversions with those of styrene. Although the polymerization of styrene reached the highest conversion (92%) after 24 h, the 4VG derivatives also achieved high conversions (Figure 7 and Table 2). It seems that the hindrance of the phenol-protecting group did not have a big impact on the final conversion. For instance, the final conversion of a monomer protected with a more bulky group such as Piv4VG was only slightly lower than the final conversion of a less hindered monomer such as Ac4VG. In general, the 4-vinyl guaiacol derivatives behaved in similar ways, reaching conversions above 75% in all cases after 24 h.

To better compare the monomers and get rid of the small experimental differences in AIBN concentration from one polymerization to another, we used the conventional radical polymerization kinetics equation to compare the homopolymerization rates for different monomers (styrene, Ac4VG, But4VG, Hept4VG). Through the *quasi-steady-state* approximation and considering the initiator concentration *[AIBN]* as constant from the beginning of the polymerization, we could use Equation (1) to calculate and compare the $\frac{k_{p}}{\sqrt{k_{t}}}$ value of styrene with those of our biobased monomers.

(1) $$ln\frac{{\left[M\right]}_{0}}{\left[M\right]}=ln\frac{1}{1-r}=k_{p}\times \sqrt{\frac{2fk_{d}[I]}{gk_{t}}}\times t$$

where *[M]* is the concentration of monomer, *k*_p_ and *k*_t_ are the propagation and termination rate constants, respectively (considering *R*_t_ = *g* × *k*_t_ × [P*]², with *g* = 2, i.e., IUPAC nomenclature), *r* is the fractional conversion of monomer, *[I]* the initial concentration of initiator, *k*_d_ is the decomposition rate constant of the initiator and *f* is the initiator efficiency coefficient.

Our experimental procedure with ^1^H-NMR online monitoring was verified with the *k*_p_ and *k*_t_ values of styrene at 70 °C which are 480 L.mol^−1^.s^−1^ and 1.2 × 10^8^ L.mol^−1^.s^−1^, respectively [49,50]. Hence, the $\frac{k_{p}}{\sqrt{k_{t}}}$ value for styrene is 0.044, very close to our experimental value of (0.056) (details into the Supporting Information).

Figure S23 and the corresponding $\frac{k_{p}}{\sqrt{k_{t}}}$ values reported in Table 2 showed a general tendency between the rates of polymerization. Even if little differences were observed in $\frac{k_{p}}{\sqrt{k_{t}}}$ values, all monomers have a value in the same order of magnitude as styrene, which makes us conclude that their kinetic behavior could be compared with styrene during conventional free radical polymerization in solution. In addition, a copolymerization experiment between Ac4VG and styrene showed similar conversion for both monomers (Figure S24).

The homopolymers were characterized by SEC (Figure S25*)*, TGA (Figure S26*)* and DSC (Figure S27) to determine the molar masses (*M*_n_) and dispersities (*Đ*), degradation temperature, and glass transition temperature (*T*_g_) (Table 2). Molar masses ranging from 7900 to 32,000 g/mol were obtained for the 4VG derivatives. 4VG was an exception, producing only oligomers of 4500 g/mol in low yield, which is consistent with its inhibiting properties. At first sight, the differences in molar masses for the homopolymers of 4VG derivatives can be quite surprising because the used polymerization conditions were the same for all homopolymers. However, since the monomer content was kept constant in the mixture (22 wt%) for a given molar quantity of monomer (7.8 mmol), the monomer concentration *[M]*_0_ and initiator concentration *[I]*_0_ in mol/L were different for each experiment, which could also explain the differences in *DP*_n_ and *M*_n_ values (*DP*_n,0_ = *R*_p,0_/*R*_t,0_ = (*k*_p_)/[(*k*_t_)^1/2^] × *[M]*_0_/[2 × (*f* × *k*_d_ × *[I]*_0_)^1/2^]).

Thermogravimetric analyses show that the biobased homopolymers have high decomposition temperatures *T*_d,5%_ > 317 °C. Only Poly4VG showed weak thermal stability (*T*_d,5%_ = 202 °C) (Figure S26) which can be due to the low molar masses of the synthesized homopolymer. In fact, this experimental decomposition temperature is lower than the value reported by Takeshima and coworkers (*T*_d,5%_ = 319 °C) [51], who were able to homopolymerize 4VG by cationic polymerization (95% of conversion after 1 h) and obtained a homopolymer with *M*_n,SEC_ = 11,800 and *Đ*= 1.28. Figure S27 shows the differential scanning calorimetry (DSC) curves and the glass transition temperature (*T*_g_) obtained for each homopolymer. The *T*_g_ measured for Poly4VG was close to the reported value (*T*_g_ = 86–92 °C) [34]. The *T*_g_ measured for Poly(Ac4VG) homopolymer was also in agreement with the reported value (110 °C) [29]. Hatekeyama and coworkers [35] described the *T*_g_ variation of Poly(Ac4VG) as a function of *M*_n_ and they found a *T*_g_ of 106 °C for samples with a molar mass of 33,000 g/mol. The experimental *T*_g_ values varied widely from 5 °C for poly(Und4VG) up to 117 °C for Poly(Ac4VG), as expected for esterified 4VG-derivatives depending on the length of the alkanoyl chain: poly(Ac4VG) (117 °C) > poly(Prop4VG) (81 °C) > poly(But4VG) (71 °C) > poly(Hept4VG) (16 °C) > poly(Und4VG) (5 °C).

It is interesting to note that an increase of two carbons in the alkyl chain caused a huge decrease of the *T*_g_ in the case of the AcHE4VG and Ac4VG: from *T*_g_ = 117 °C for poly(Ac4VG) down to 37 °C for poly(AcHE4VG). This result was also in accordance with the *T*_g_ value reported by Alexakis and coworkers, who reported for poly(methyl 2-(2-methoxy-4-vinylphenoxy)acetate)) (the intermediate between Ac4VG and AcHE4VG, containing only one methylene spacer -CH_2_-) a *T*_g_ of 46 °C [38]. In addition, we attempted to calculate the *T*_g,calc_ values by using the method of additive group contributions proposed by van Krevelen et al. [52] (see the Supporting Information for the detailed calculations). Interestingly, the trend that is experimentally observed for the *T*_g,exp_ values is in good agreement with the trend of the calculated *T*_g,calc_ values (Table 2). Furthermore, except for poly(Und4VG) for which the calculated value *T*_g,calc_ = −35 °C differed significantly from the experimental value *T*_g,exp_ = 5 °C, the calculated values for most homopolymers of 4VG derivatives were close to the experimental values (|*T*_g,exp_ − *T*_g,calc_| < 10 °C). The wide range of *T*_g_ values obtained for these new biobased homopolymers not only allows substituting styrene by Ac4VG in the case of high *T*_g_, but also opens the door to applications in soft low *T*_g_ materials.

## 3. Experimental Section

### 3.1. Materials

4-vinyl guaiacol (4VG, 99%, AABlocks, San Diego, CA, USA), acetic anhydride (99%, Aldrich, St. Louis, MO, USA), sodium acetate (AcONa, ≥99%, Aldrich), sodium propionate (≥99%, Aldrich), propionic anhydride (≥99%, Aldrich), N,N’-dicyclohexylcarbodiimide (DCC, ≥99%, Aldrich), 4-dimethylaminopyridine (DMAP, ≥99%, Aldrich), propionic acid (99.5%, Aldrich), dichloromethane (CH_2_Cl_2_, 99.9%, Aldrich), butyric acid (99%, Aldrich), butyric anhydride (98%, Aldrich), sodium butyrate (98%, Aldrich), pivalic acid (99%, Aldrich), sodium pivalate (≥95%, BLD Pharmatech, Kaiserslautern, Germany), pivalic anhydride (99%, Aldrich), ethylene carbonate (98%, Aldrich), 1,5-diazabicyclo[4.3.0]non-5-ene (DBN, 98%, Aldrich), sodium hydroxide (NaOH, 98%, VWR, Rosny-sous-Bois, France), hydrochloric acid (HCl, 37%, Alfa-Aesar, Karlsruhe, Germany), iodomethane (≥99%, Aldrich), acetone (99%, Aldrich), potassium carbonate (99%, Aldrich), 1,4-bis(trimethylsilyl)benzene (96%, Aldrich), heptanoic acid (99%, Aldrich), undecanoic acid (98%, Aldrich), and tetrahydrofuran (THF, technical grade, Aldrich) were used as received. 2,2′-Azobis(2-methylpropionitrile) (AIBN, 98%Fluka, Seelze, Germany) was recrystallized in methanol (MeOH, 99.8%, Aldrich) and dried at room temperature under vacuum (10^−3^ mbar) for 2 h before use.

### 3.2. Methods

#### 3.2.1. Synthesis of 2-Methoxy-4-vinylphenyl Acetate (Ac4VG)

In a double-necked round-bottom flask equipped with a condenser, a mixture of 4VG (12 mmol, 1.8 g, 1 eq) and sodium acetate (0.5 mmol, 41 mg, 0.04 eq) was dissolved in acetic anhydride (15 mmol, 1.5 g, 1.25 eq), and stirred with a magnetic stir bar at 90 °C for 30 min under air (*conversion*_4VG_ = 100%). In total, 5 mL of ethyl acetate was then added to the reaction mixture, which was then washed with a saturated brine solution (3 × 5.0 mL). The organic phase was washed with an aqueous phase acidified with a 1.0 M HCl solution to a pH of 2, and the organic phase was isolated and dried with Na_2_SO_4_. The solvent was removed at 30 °C under vacuum to afford the crude product as a yellowish liquid. *Gravimetric yield*: 95%.

#### 3.2.2. Synthesis of 2-Methoxy-4-vinylphenyl Propionate (Prop4VG)

Chemical Route 1: In a double-necked round-bottom flask equipped with a condenser, a mixture of 4VG (1.8 g, 12 mmol, 1 eq) and sodium propionate (0.48 g, 5 mmol, 0.41 eq) was dissolved in propionic anhydride (1.56 g, 12 mmol, 1 eq), and stirred at 90 °C for 30 min under air (*conversion*_4VG_ = 100%). In total, 5 mL of ethyl acetate was then added to the reaction mixture, which was then washed with a saturated brine solution (3 × 5.0 mL). The organic layer was washed once with a 1.0 M HCl solution and three times with a saturated NaHCO_3_ solution. The solvent was removed under vacuum at room temperature. The crude product was isolated as a colorless oil. *Gravimetric yield*: 54%.

Chemical Route 2: In a double-necked round-bottom flask equipped with a condenser, DCC (2.7 g, 13.09 mmol, 1.1 eq) and DMAP (0.36 g, 2.975 mmol, 0.25 eq) were added to a solution of propionic acid (0.98 g, 12 mmol, 1 eq) and 4VG (1.8 g, 12 mmol, 1 eq) in CH_2_Cl_2_ (38 mL) under N_2_ flow. The reaction mixture was stirred for 3 h at 50 °C and 18 h at room temperature (*conversion*_4VG_ = 95%). The mixture was filtered over Buchner to remove the solids. The resulting DCM solution was washed with saturated NaHCO_3_ solution (2 × 20 mL). The combined aqueous fractions were washed with CH_2_Cl_2_ (20 mL × 2). The combined organic fractions were washed with brine (25 mL × 1) and dried over Na_2_SO_4_. The solvent was removed under vacuum at room temperature. The crude product was isolated as a colorless oil. *Gravimetric yield*: 85%.

#### 3.2.3. Synthesis of 2-Methoxy-4-vinylphenyl Butyrate (But4VG)

Chemical Route 1: In a double-necked round-bottom flask equipped with a condenser, a mixture of 4VG (5 g, 33.3 mmol, 1 eq) and sodium butyrate (0.37 g, 3.33 mmol, 0.1 eq) was dissolved in butyric anhydride (5.27 g, 33.3 mmol, 1 eq), and stirred at 90 °C for 30 min under air (*conversion*_4VG_ = 97%). In total, 5 mL of ethyl acetate was added to the reaction mixture, which was then washed with a saturated brine solution (3 × 5.0 mL). The organic layer was washed once with a 1.0 M HCl solution and three times with a saturated NaHCO_3_ solution. The solvent was removed under vacuum at room temperature. The crude product was isolated as a colorless oil. *Gravimetric yield*: 82%.

Chemical Route 2: In a double-necked round-bottom flask surmounted with a condenser, DCC (2.7 g, 13.09 mmol, 1.1 eq) and DMAP (0.36 g, 2.975 mmol, 0.25 eq) were added to a solution of butyric acid (1.04 g, 11.9 mmol, 1 eq) and 4VG (1.8 g, 11.9 mmol, 1 eq) in CH_2_Cl_2_ (38 mL) under N_2_ flow. The reaction mixture was stirred for 3 h at 50 °C and 18 h at room temperature (*conversion*_4VG_ = 96%). The mixture was filtered on Buchner to remove the solids. The resulting DCM solution was washed with saturated NaHCO_3_ solution (2 × 20 mL). The combined aqueous fractions were washed with CH_2_Cl_2_ (20 mL × 2). The combined organic fractions were washed with brine (25 mL × 1) and dried over Na_2_SO_4_. The solvent was removed under vacuum at room temperature. The crude product was isolated as a colorless oil. *Gravimetric yield*: 99%.

#### 3.2.4. Synthesis of 2-Methoxy-4-vinylphenyl Pivalate (Piv4VG)

Chemical Route 1: In a double-necked round-bottom flask equipped with a condenser, a mixture of 4VG (1.5 g, 10 mmol, 1 eq) and sodium pivalate (0.12 g, 1 mol, 0.1 eq) was dissolved in pivalic anhydride (1.86 g, 10 mmol, 1 eq), and stirred at 90 °C for 30 min under air (*conversion*_4VG_ = 92%). In total, 5 mL of ethyl acetate was added to the reaction mixture, which was washed three times with a saturated NaHCO_3_ solution and once with distilled water. The solvent was removed under vacuum at room temperature. The crude product was isolated as a pale yellow oil. *Gravimetric yield*: 68%.

Chemical Route 2: In a double-necked round-bottom flask surmounted with a condenser, DCC (2.7 g, 13.09 mmol, 1.1 eq) and DMAP (0.36 g, 2.975 mmol, 0.25 eq) were added to a solution of pivalic acid (1.21 g, 11.9 mmol, 1 eq) and 4VG (1.8 g, 11.9 mmol, 1 eq) in CH_2_Cl_2_ (38 mL) under N_2_ flow. The reaction mixture was stirred for 3 h at 50 °C and 18 h at room temperature (*conversion*_4VG_ = 67%). The mixture was filtered on Buchner to remove the solids. The resulting DCM solution was washed with saturated NaHCO_3_ solution (2 × 20 mL). The combined aqueous fractions were washed with CH_2_Cl_2_ (20 mL × 2). The combined organic fractions were washed with brine (25 mL × 1) and dried over Na_2_SO_4_. The solvent was removed under vacuum at room temperature. The crude product was recovered as a pale yellow oil. *Gravimetric yield*: 51%.

#### 3.2.5. Synthesis of 2-Methoxy-4-vinylphenyl Heptanoate (Hept4VG)

In a double-necked round-bottom flask surmounted with a condenser, DCC (15.11 g, 73.26 mmol, 1.1 eq) and DMAP (0.81 g, 6.66 mmol, 0.1 eq) were added to a solution of heptanoic acid (10.4 g, 80 mmol, 1 eq) and 4VG (10 g, 66.6 mmol, 1 eq) in CH_2_Cl_2_ (211 mL) under N_2_ flow. The reaction mixture was stirred for 3 h at 50 °C and 18 h at room temperature (*conversion*_4VG_ = 94%). The mixture was filtered on Buchner to remove the solids. The resulting DCM solution was washed with saturated NaHCO_3_ solution (2 × 50 mL). The combined aqueous fractions were washed with CH_2_Cl_2_ (20 mL × 2). The combined organic fractions were washed with brine (50 mL × 1) and dried over Na_2_SO_4_. The solvent was removed under vacuum at room temperature. *Gravimetric yield*: 76%. In order to eliminate any residual 4VG that could inhibit the radical polymerization, the product was subsequently purified by column chromatography (cyclohexane:ethyl acetate/9:1). Final purity by ^1^H NMR: 100%. The crude product was isolated as a colorless oil.

#### 3.2.6. Synthesis of 2-Methoxy-4-vinylphenyl Undecanoate (Und4VG)

In a double-necked round-bottom flask surmounted with a condenser, DCC (7.55 g, 36.6 mmol, 1.1 eq) and DMAP (0.4 g, 3.33 mmol, 0.1 eq) were added to a solution of undecanoic acid (6.2 g, 33.3 mmol, 1 eq) and 4VG (5 g, 33.3 mmol, 1 eq) in CH_2_Cl_2_ (110 mL) under N_2_ flow. The reaction mixture was stirred for 3 h at 50 °C and 18 h at room temperature (*conversion*_4VG_ = 94%). The mixture was filtered on Buchner to remove the solids. The resulting DCM solution was washed with saturated NaHCO_3_ solution (2 × 50 mL). The combined aqueous fractions were washed with CH_2_Cl_2_ (20 mL × 2). The combined organic fractions were washed with brine (50 mL × 1) and dried over Na_2_SO_4_. The solvent was removed under vacuum at room temperature. *Gravimetric yield*: 98%. In order to eliminate any residual 4VG that could inhibit the radical polymerization, the product was subsequently purified by column chromatography (cyclohexane:ethyl acetate/9:1). Final purity by ^1^H NMR: 100%. The crude product was isolated as a colorless oil.

#### 3.2.7. Synthesis of 2-(2-Methoxy-4-vinylphenoxy)ethan-1-ol (HE4VG)

4-vinyl guaiacol (1.5 g, 10 mmol, 1 eq) and ethylene carbonate (0.97 g, 11 mmol, 1.1 eq) were placed in a two-necked round-bottom flask surmounted with a condenser and mixed under N_2_ atmosphere and high magnetic stirring. The flask was then immersed in an oil bath set to 150 °C. Once the ethylene carbonate had completely melted and the reaction mixture was homogeneous, DBN (0.62 mL, 0.5 mmol, 0.005 eq) was injected slowly into the reaction mixture via a syringe. The reaction proceeded at 150 °C for 30 min (*conversion*_4VG_ = 98%). The product was dissolved in DCM and extracted 3 times with 1 M HCl, to remove any residues of ethylene carbonate and DBN. The organic phase was dried with Na_2_SO_4_ and the solvent was evaporated. The crude product was isolated as a white solid. *Gravimetric yield*: 73%.

#### 3.2.8. Synthesis of 2-(2-Methoxy-4-vinylphenoxy)ethyl Acetate (AcHE4VG)

In a double-necked round-bottom flask equipped with a condenser, a mixture of hydroxyethyl4VG (0.37 g, 1.9 mmol, 1 eq) and sodium acetate (0.0075 g, 0.095 mmol, 0.05 eq) was dissolved in acetic anhydride (0.24 g, 2.4 mmol, 1.25 eq) and stirred at 90 °C for 30 min under air (*conversion*_HE4VG_= 100%). In total, 5 mL of ethyl acetate was added to the reaction mixture and washed with a saturated brine solution (3 × 5.0 mL). The organic phase was washed with an aqueous phase acidified with a 1.0 M HCl solution to a pH of 2, the organic phase was isolated and dried with Na_2_SO_4_. The solvent was removed under vacuum at room temperature. The crude product was isolated as a yellow oil. *Gravimetric yield*: 35%.

#### 3.2.9. Synthesis of 1,2-Dimethoxy-4-vinylbenzene (Met4VG)

Potassium carbonate (6.8 g, 49.2 mmol, 4.9 eq) was added to a flask with 50 mL of acetone and 4-vinyl guaiacol (1.5 g, 10 mmol, 1 eq) followed by iodomethane (14.7 g, 104 mmol, 10.4 eq). After reflux under N_2_ at 55 °C for 6h (*conversion*_4VG_ = 96%), the solution was filtered on Buchner and concentrated under vacuum at room T. *Gravimetric yield*: 98%.

#### 3.2.10. General Procedure for the Radical Homopolymerization of 4-vinyl Guaiacol Derivatives in Toluene

4-vinyl guaiacol derivative (7.8 mmol, 1 eq), 1,4-bis(trimethylsilyl)benzene (0.05 eq) as internal standard and toluene were placed in a double-necked flask. The flask was sealed with a septum and the reaction mixture was purged with N_2_ bubbling for 30 min. The reaction mixture was placed in an oil bath at 70 °C under magnetic stirring. AIBN (1.3 wt% based on monomer) previously dissolved in toluene (2 g) and purged with N_2_ for 10 min was added to the reaction mixture to start the polymerization. The amount of toluene was calculated to obtain a monomer content in the reaction mixture of 22 wt%. The monomer conversion was followed by ^1^H NMR. The conversion was determined every 30 min for the first 2 h of reaction, then every 1 h until 8 h of reaction and then measured after 24 h reaction. The crude reaction mixture was diluted in a minimum amount of toluene and added dropwise into cold MeOH (50 mL) under vigorous stirring. The resulting suspension was stirred for 2 h, then the precipitate was decanted, the serum was removed and the precipitate was washed two more times with 30 mL of MeOH to remove any residual 4VG derivative. The solvent was evaporated under reduced pressure (10^−2^ mbar) in an oven at 40 °C for 24 h.

#### 3.2.11. General Procedure for the Radical Homopolymerization of Styrene and 4-vinyl Guaiacol Derivatives in d_8_-toluene (Online ^1^H-NMR Measurements)

In a typical procedure, styrene (7.8 mmol, 1 eq), 1,4-bis(trimethylsilyl)benzene (0.05 eq) as internal standard, AIBN (1.3 wt% based on monomer) and d_8_-toluene were placed in a round bottom flask. The amount of d_8_-toluene was calculated to obtain a monomer content in the reaction mixture of 22 wt%. The mixture was stirred for 5 min to completely homogenize the reaction medium. In total, 3 mL of the mixture was placed in a 5 mm diameter NMR tube and purged with N_2_ for 2 min. The monomer conversion was followed by online ^1^H-NMR measurements with a Bruker 400 MHz spectrometer (16 scans, d_8_-toluene) previously equilibrated at 70 °C.

### 3.3. Characterization

#### 3.3.1. Thermogravimetric Analysis

Thermogravimetric analysis (TGA) was performed on 10–15 mg dry polymer samples on a TA Q50 Instruments (Gayancourt, France) from 20 °C to 590 °C, in an aluminum pan, at a heating rate of 20 °C min^−1^, under nitrogen.

#### 3.3.2. Differential Scanning Calorimetry

Differential scanning calorimetry (DSC) measurements were performed on 10−15 mg samples, under a nitrogen atmosphere, with a Netzsch DSC 200 F3 instrument (Selb, Germany) using a heating/cooling cycle adapted to each analysis. For the melting point (monomer analyses), three cycles of heating/cooling from −100 to 100 °C at 10 °C/min were carried out. The reported results were extracted from the third heating ramp. For glass transition temperature (dry homopolymer analyses), three cycles of heating/cooling from 20 to 170 °C at 20 °C/min for high *T*_g_ polymers and three cycles of temperature rising from −60 to 60 °C at 20 °C/min for low *T*_g_ polymers were carried out. The reported results were extracted from the second heating ramp.

#### 3.3.3. Size Exclusion Chromatography

Size exclusion chromatography (SEC) from Agilent Technologies model 1260 Infinity (1260 Iso Pump) (Waldbronn, Germany), with its corresponding Agilent GPC/SEC software version 1.2, was used, equipped with two PL1113−6300 ResiPore 300 × 7.5 mm columns (up to 500,000 g mol^−1^) thermostated at 35 °C, and refractive index (RI) detector (Varian 390-LC) (Church Stretton, United Kingdom). Calibration was performed with PMMA narrow standards (molar masses between 2,210,000 and 100 g/mol). THF was used as the eluent at a flow rate of 1 mL min^−1^. The typical sample concentration was 10 mg mL^−1^ and it was analyzed on the crude reaction medium diluted in THF (before the polymer purification).

#### 3.3.4. Nuclear Magnetic Resonance Spectroscopy

^1^H-NMR spectroscopy was performed with a Bruker Avance 400 MHz spectrometer (Fällanden, Switzerland) at room temperature. The spectra were recorded by dissolving 0.1 mL of samples in 0.5 mL of CDCl_3._

#### 3.3.5. Gas Chromatography

Gas chromatography coupled to mass spectrometry (GC-MS) was performed with a QP2010 SE Shimadzu chromatographer (Canby, OR, USA) surmounted with a Zebron ZB-5ms column (30 m × 0.25 mm × 0.25 µm). The carrier gas was He, the column flow was set at 1 mL/min and the injection mode was split (ratio 1:30). The injector temperature was set at 250 °C. The GC detector was composed of a quadrupole system and the MS detector was an electronic impact. The oven was set at 50 °C for 2 min and then the temperature was raised to 280 °C at 22 °C/min. The typical sample concentration was 1 mg in 2 mL of CH_2_Cl_2_.

## 4. Conclusions

In this work, we showed that 4-vinyl guaiacol (4VG) is a suitable fully biobased molecule to develop a versatile platform of biobased aromatic monomers. 4VG was functionalized to prepare nine biobased monomers; seven of them had never been synthesized before. The considered synthesis procedures were investigated from a sustainable point of view taking into account the raw materials sustainability and the process green metrics. The synthesized biobased monomers were successfully polymerized via conventional radical polymerization in solution. Lower polymerization rates and plateauing of monomer conversion were observed when 4VG was present in the reaction medium. Additionally, the homopolymers showed a large range of thermochemical properties, in particular permitting access to a wide range of *T*_g_ (from 117 °C down to 5 °C) depending on the length of the alkyl chain in the ester or ether groups. Through this work, we generated a catalog of biobased aromatic monomers able to react by radical polymerization. The new monomers could be employed in copolymerization aiming at producing new biobased polymers and materials either as alternatives to their petroleum-based counterparts or as new materials with differentiated properties.

## Figures and Tables

**Table 1:** Green metrics of 4VG derivatives ^a^.

	Esterification										Etherification	
Name	Ac4VG	Prop4VG		But4VG		Piv4VG		Hept4VG	Und4VG	AcHE4VG	HE4VG	Met4VG
		Route 1	Route 2	Route 1	Route 2	Route 1	Route 2					
*AE* (%)	76	73	91	71	92	69	92	93	95	80	81	56
*E-factor* (kg waste/kg product)	0.5	1.9	19.9	0.8	15.8	1.2	30.2	17.6	11.4	2.9	1.2	44.4
*C-efficiency* (%)	74	40	36	61	45	55	24	38	56	28	51	40
*RME* (%)	66	35	36	56	44	45	23	37	54	26	46	10
*Biobased carbon* (%)	82	75	100	69	100	64	64	100	100	69	82	90

^a^ The typical formulae used for the calculations are reported in the Supporting Information (*AE*: atom economy, *RME*: reaction mass efficiency) and the details of the calculation are reported in Table S1.

**Table 2:** Summary of homopolymers properties.

Monomer	Monomer Conversion (%) (24 h)	$\frac{k_{p}}{\surd k_{t}}$	*T*_d,5%_ (°C) ^a^	*T*_g,exp_ (°C) ^a^	*T*_g,calc_ (°C) ^b^	*M*_n_ (g/mol) (24 h) ^c^	*Đ* (24 h) ^c^
Styrene	97	0.056	365	104	100	7,500	1.7
4VG	21	n.m.	202	83	n.d.	4,500	2.4
Ac4VG	88	0.123	317	117	110	14,700	2.3
Prop4VG	86	n.m.	341	81	84	13,900	2.3
But4VG	95	0.106	346	71	62	15,200	2.4
Piv4VG	81	n.m.	346	96	102	32,000	1.9
Hept4VG	88	0.153	344	16	11	23,700	2.3
Und4VG	81	n.m.	332	5	−35	7900	2.4
HE4VG	84	n.m.	n.m. ^d^	n.m.	n.d.	n.m.	n.m.
AcHE4VG	95	n.m.	334	37	38	11,600 and 7300 (bimodal)	1.4 and 1.9
Met4VG	76	n.m.	357	71	78	11,900	1.7

^a^ *T*_d,5%_ and *T*_g_ on dried samples after precipitation in methanol. ^b^ *T*_g,calc_ was calculated using the method of additive group contributions. ^c^ *M*_n_ and dispersity on crude reaction medium; SEC calibration was performed with PMMA narrow standards. ^d^ We were not able to purify PolyHE4VG by precipitation in MeOH; hexane might be a more appropriate solvent to precipitate this polymer. n.m. stands for not measured. n.d. stands for not determined.

## Data Availability

Data are contained within the article and Supplementary Materials.

## Supplementary Materials

The following supporting information can be downloaded at: https://www.mdpi.com/article/10.3390/molecules29112507/s1. Formulae used in the article; kinetics of polymerization; charaterizations of the monomers; calculation of green metrics; results of homopolymerization; results of copolymerization; characterizations of the homopolymers. References [53,54,55,56,57,58,59,60] are cited in the Supplementary Materials.
