# Supplementary material for: 4-Vinyl Guaiacol: A Key Intermediate for Biobased Polymers
Source: Molecules. 2024 May 25;29(11):2507. doi: 10.3390/molecules29112507 (PMC11174018; doi:10.3390/molecules29112507)
Supplement: Supplementary file 1 [file molecules-29-02507-s001.zip › molecules-2948350-supplementary.pdf]

Supporting Information

4-Vinyl Guaiacol: A Key Intermediate for Biobased Polymers

Elena Rigo,[1,2,3] Cédric Totée,[1] Vincent Ladmiral,[1] Sylvain Caillol[1] and Patrick Lacroix-Desmazes[1,]*

[1] ICGM, University of Montpellier, CNRS, ENSCM, 34293 Montpellier, France.

[2] Synthomer Speciality Chemicals SAS, 76430 Sandouville, France.

[3] Synthomer Ltd., Harlow CM20 2BH, United Kingdom.

[*] patrick.lacroix-desmazes@enscm.fr

Formulae used in the article:

Monomer syntheses:

Gravimetric yield = $\frac{\boldsymbol{m\,(exp)}}{\boldsymbol{m(theo)}} \times \mathbf{100} \qquad (\boldsymbol{equation\ S1})$

4VG conversion (%) was calculated from the integrations of ^{1}H NMR spectra.

$$Conversion_{4VG}\ (\%) = \frac{\frac{\int \text{desired product}}{\text{nb of protons}}}{\frac{\int \text{desired product}}{\text{nb of protons}} + \frac{\int 4VG}{nb\ of\ protons}} \times \mathbf{100} \qquad (\boldsymbol{equation\ S2})$$

With the hypothesis that all the 4VG is converted into monomer, no secondary reactions take place.

Green metrics:

- Atom economy: measure of the amount of starting materials that end up as useful products.

$$\boldsymbol{Atom\ economy}\ (\%) = \frac{\boldsymbol{Molecular\ Weight\ (MW)\ of\ desired\ product}}{\sum \boldsymbol{MW\ \ of\ reactants}} \times \mathbf{100} \quad (\boldsymbol{equation\ S3})$$

- E-factor: measure of the amount of waste produced in a process.

$$\boldsymbol{E - Factor} = \frac{\boldsymbol{m\ of\ total\ waste}}{\boldsymbol{m\ of\ desired\ product}} \qquad (\boldsymbol{equation\ S4})$$

- Carbon efficiency: measure of the amount of carbon atoms in the starting materials that end up in the desired product.

$$\boldsymbol{Carbon\ efficiency}\ (\%) = \frac{\sum \boldsymbol{m\ of\ C\ in\ the\ desired\ product}}{\sum \boldsymbol{m\ \ of\ C\ of\ reactants}} \times \mathbf{100} \quad (\boldsymbol{equation\ S5})$$

- Reaction mass efficiency: measure of the efficiency with which reactant mass ends up in the desired product.

$$\boldsymbol{Reaction\ mass\ efficiency}\ (\%) = \frac{\boldsymbol{m\ of\ desired\ product}}{\sum \boldsymbol{m\ of\ reactants}} \times \mathbf{100}\ (\boldsymbol{equation\ S6})$$

- Biobased carbons: measure of the amount of renewable carbon in the final monomer.

$$\boldsymbol{Biobased\ carbon}\ (\%) = \frac{\sum \boldsymbol{biobased\ atoms\ of\ C}}{\sum \boldsymbol{total\ atoms\ of\ C}} \times \mathbf{100}\ (\boldsymbol{equation\ S7})$$

Kinetics of polymerization:

Monomer conversion in radical homopolymerization of 4-vinyl guaiacol derivatives in toluene (disappearance of the signals representing the reactive double bond) was determined by ^{1}H-NMR analysis:

- Calculation with the internal standard (IS) (1,4-bis(trimethylsilyl)benzene) (signal of the IS around 0.5 ppm):

$$\boldsymbol{monomer\ conversion}\ (\%) = \mathbf{100} \times \left(\mathbf{1} - \left(\frac{\frac{\boldsymbol{Monomer\ signal\ integration}_{t=x}\ (\mathbf{5.39}\ \boldsymbol{ppm})}{\boldsymbol{IS\ integration}_{t=x}\ (\mathbf{0.43}\ \boldsymbol{ppm})}}{\frac{\boldsymbol{Monomer\ signal\ integration}_{t=0}(\mathbf{5.39}\ \boldsymbol{ppm})}{\boldsymbol{IS\ integration}_{t=0}\ (\mathbf{0.43}\ \boldsymbol{ppm})}}\right)\right) (\boldsymbol{equation\ S8})$$

Example (from ***Figure S1*** and ***S2***):

$$\boldsymbol{monomer\ conversion}\ (\%) = \mathbf{100} \times \left(\mathbf{1} - \left(\frac{\frac{\mathbf{0.415}}{\mathbf{1}}}{\frac{\mathbf{0.815}}{\mathbf{1}}}\right)\right) = \mathbf{49\%}$$

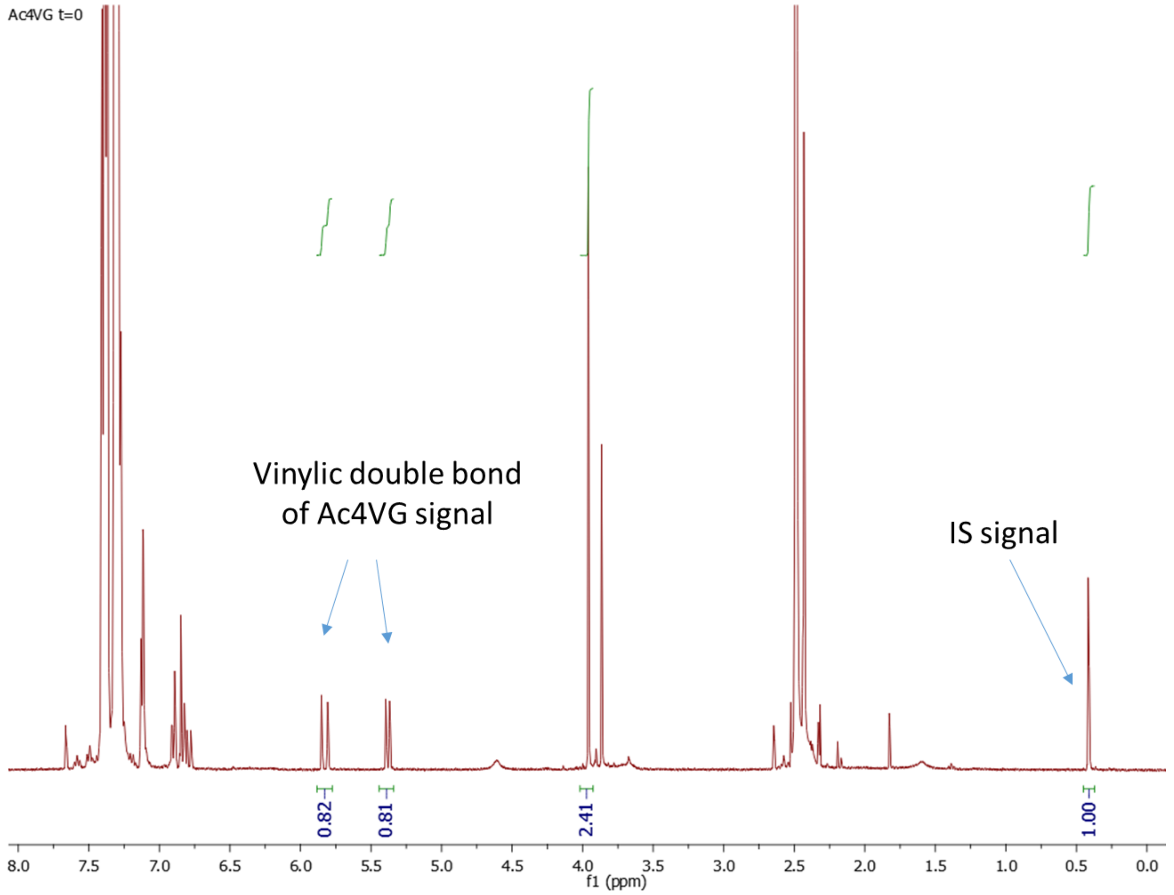

Figure S 1: *^{1}H NMR Ac4VG solution homopolymerization t=0.*

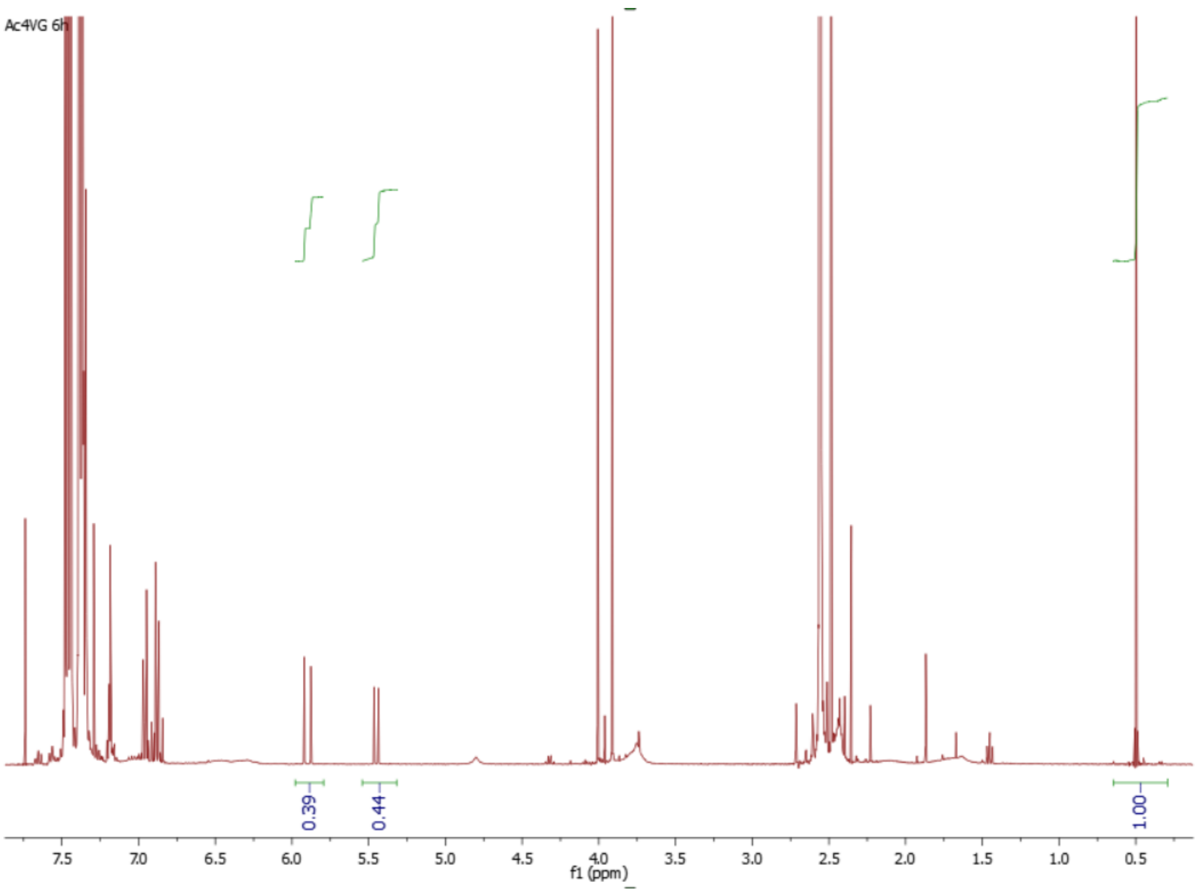

Figure S 2: *[1] H NMR Ac4VG solution homopolymerization t=6h.*

Monomers characterizations

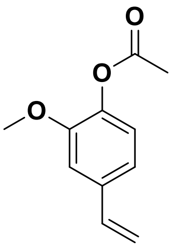

Acronym: Ac4VG
IUPAC Name: 2-methoxy-4-vinylphenyl acetate
Chemical Formula: $C_{11}H_{12}O_3$
Molecular Weight: 192.08 g/mol
Appearance: Yellow oil

^{1}H NMR (400 MHz, $CDCl_3$) : 7.26ppm solvent ($CDCl_3$), 6.99ppm (m, 3H, **H**-Ph), 6.68ppm (dd, 1H, C**H**=CH_2), 5.70ppm (d, 1H, CH=CH**Htrans**), 5.25ppm (d, 1H, CH=C**Hcis**H), 4.13 ppm (solvent, ethyl acetate), 3.85ppm (s, 3H, -OC**H₃**), 2.31 ppm (s, 3H, -C**H₃**) 2.05 ppm (solvent, ethyl acetate), 1.26 ppm (solvent, ethyl acetate).

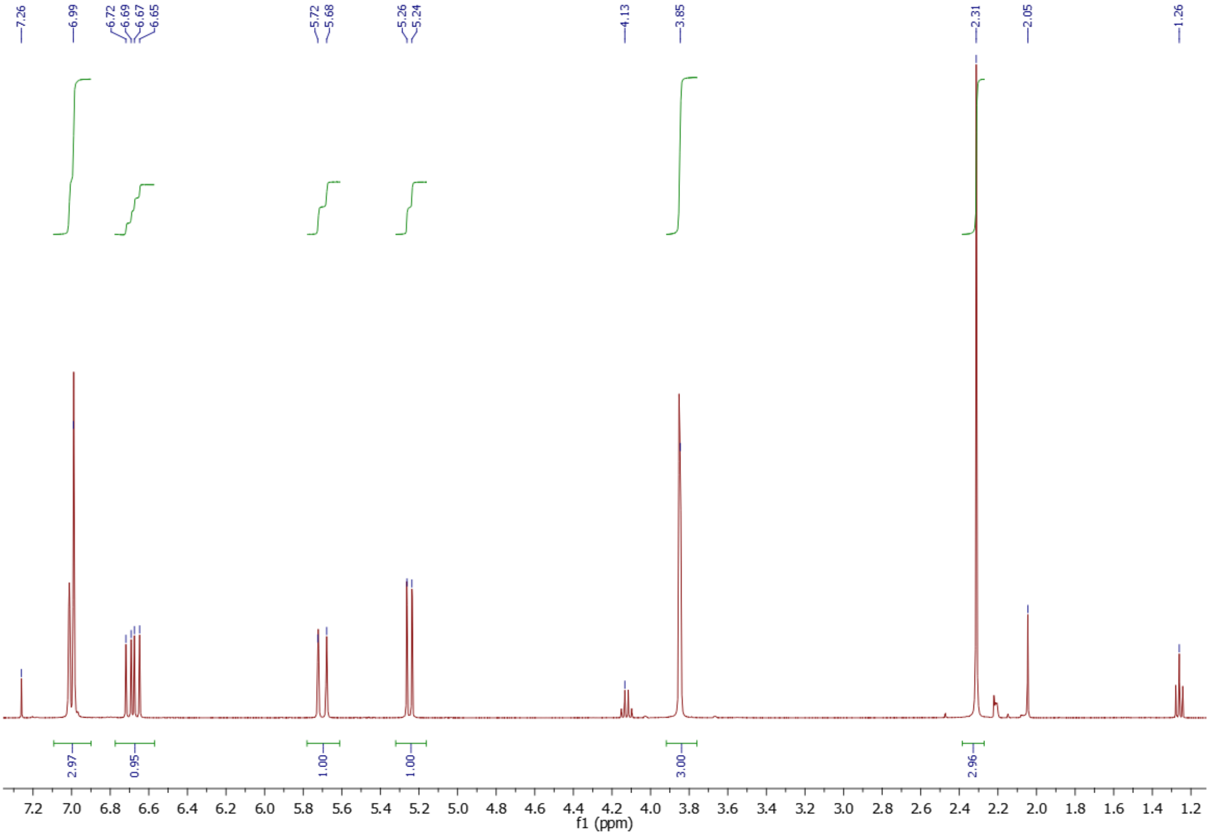

Figure S 3: *^{1}H NMR of Ac4VG in $CDCl_3$.*

GC-MS: 100% purity, only one signal (8.18 min) with MW: 192 g/mol representing Ac4VG.

Sample Information

Sample Name	: ER007
Vial #	: 20
Injection Volume	: 1.00
Data File	: C:\GCMSsolution\Data\Project1\IAM\Elena RIGO\200422-ER007.qgd
Method File	: C:\GCMSsolution\Data\Project1\IAM\Elena RIGO\50-280 (split 30) début 2.5min + g
Tuning File	: C:\GCMSsolution\System\Tune1\2022.01.04.qgt

Chromatogram ER007 C:\GCMSsolution\Data\Project1\IAM\Elena RIGO\200422-ER007.qgd

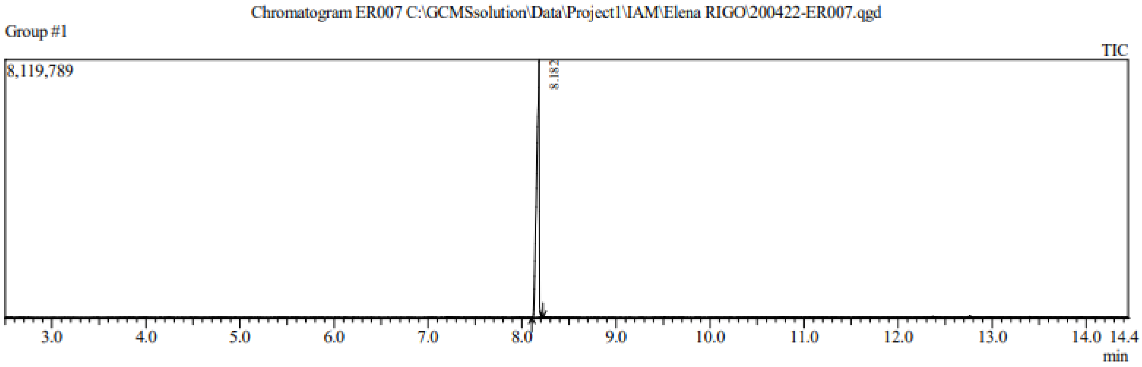

Peak Report

Peak#	R.Time	I.Time	F.Time	Area	Area%	Height	Name
1	8.182	8.103	8.223	17136007	100.00	8069511	4-Acetoxy-3-methoxystyrene
				17136007	100.00	8069511	

Spectrum

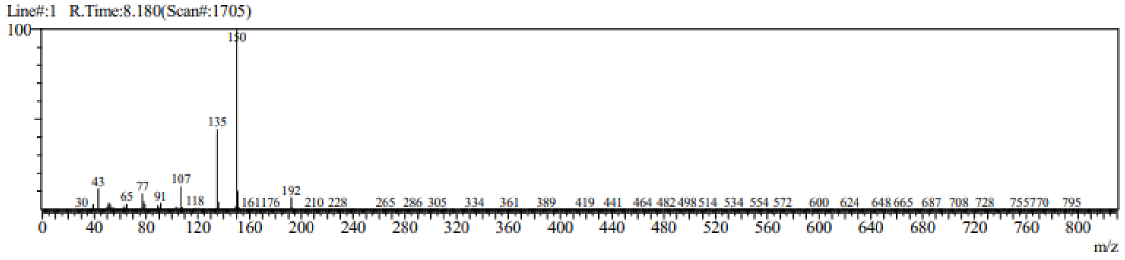

Figure S 4 : *GC chromatogram and MS spectrum of Ac4VG.*

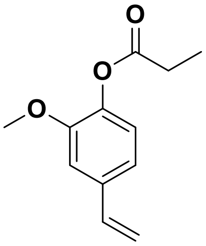

Acronym: Prop4VG
IUPAC Name: 2-methoxy-4-vinylphenyl propionate
Chemical Formula: $C_{12}H_{14}O_3$
Molecular Weight: 206.09 g/mol
Appearance: Transparent oil

^{1}H NMR (400 MHz, $CDCl_3$) : 7.26ppm solvent ($CDCl_3$), 7ppm (m, 3H, **H**-Ph), 6.68ppm (dd, 1H, C**H**=CH_2), 5.69ppm (d, 1H, CH=CH**Htrans**), 5.24ppm (d, 1H, CH=C**Hcis**H), 3.85ppm (s, 3H, -OC**H**$_3$), 2.61ppm (q, 2H, OCOC**H$_2$**CH_3), 1.56ppm (water, impurity), 1.27ppm (t, 3H, OCOCH_2C**H$_3$**), 1.20 ppm (solvent, ethyl acetate).

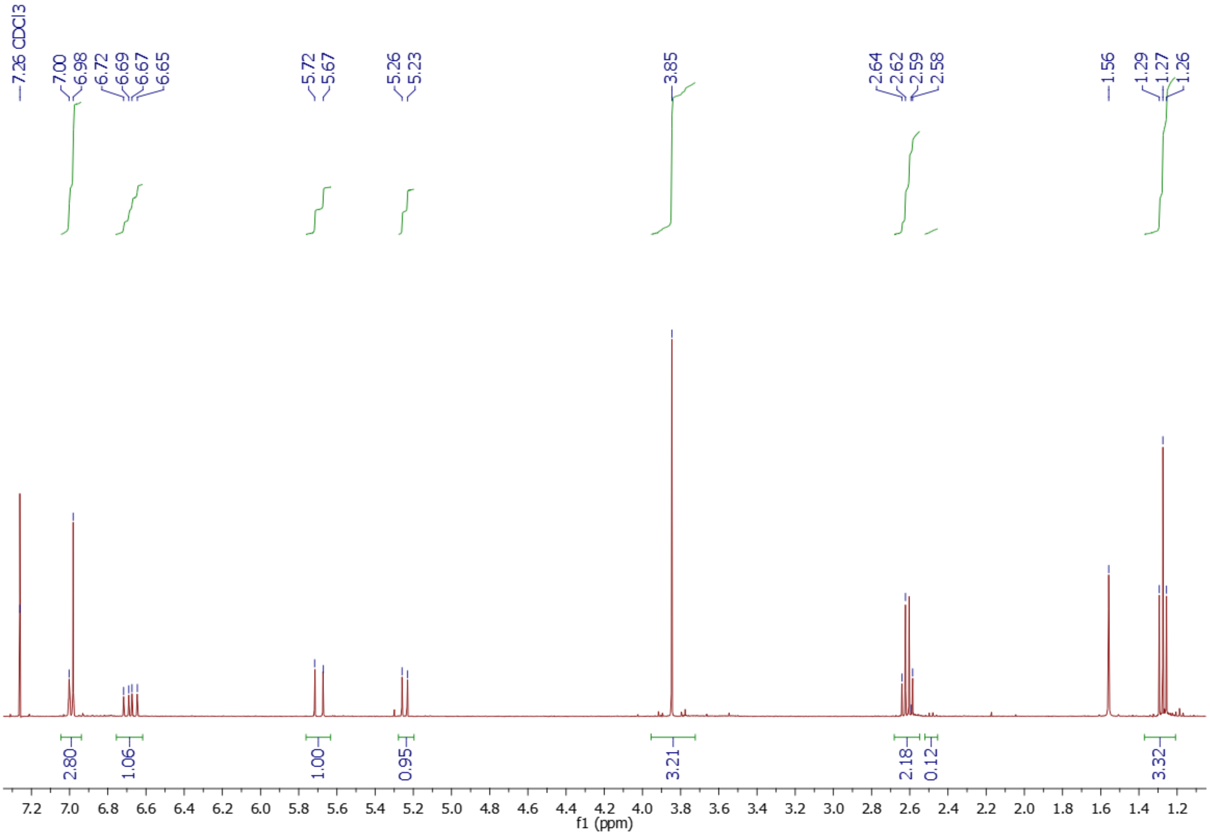

***Figure S 5:** 1H NMR of Prop4VG in CDCl3.*

GC-MS: 100% purity, only one signal (8.70 min) with MW: 206 g/mol representing Prop4VG.

Sample Information

Sample Name	: ER028
Vial #	: 24
Injection Volume	: 1.00
Data File	: C:\GCMSsolution\Data\Project1\IAM\Elena RIGO\220707_ER028ok.qgd
Method File	: C:\GCMSsolution\Data\Project1\IAM\Elena RIGO\50-280 (split 30) début 2.5min + g
Tuning File	: C:\GCMSsolution\System\Tune1\2022.06.28.qgt

Chromatogram ER028 C:\GCMSsolution\Data\Project1\IAM\Elena RIGO\220707_ER028ok.qgd

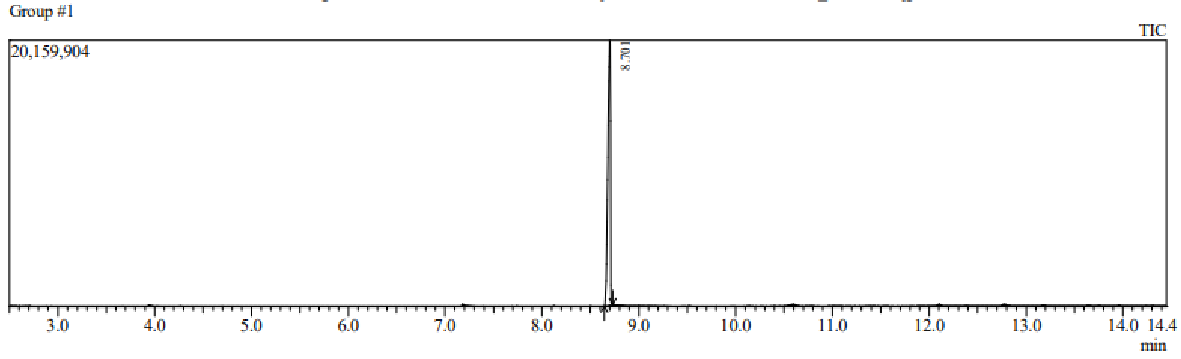

Peak Report

Peak#	R.Time	I.Time	F.Time	Area	Area%	Height	Name
1	8.701	8.643	8.730	36972233	100.00	20025903	Prop4VG (MW=206 g/mol)
				36972233	100.00	20025903	

Spectrum

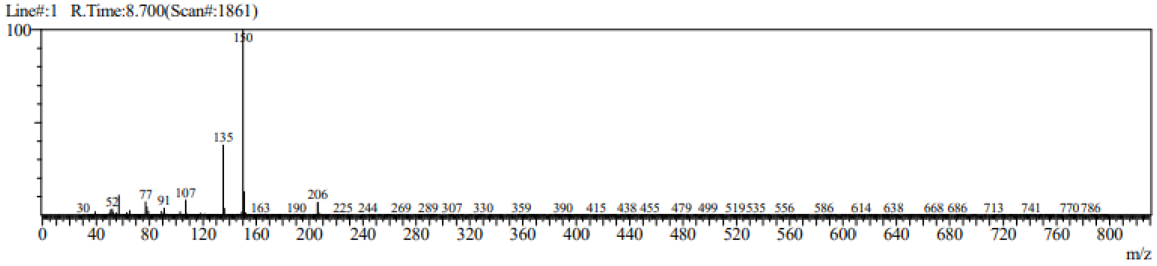

Figure S 6 : *GC chromatogram and MS spectrum of Prop4VG.*

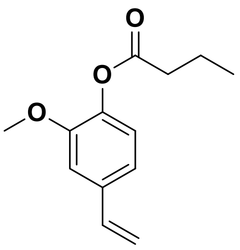

Acronym: But4VG
IUPAC Name: 2-methoxy-4-vinylphenyl butyrate
Chemical Formula: $C_{13}H_{16}O_3$
Molecular Weight: 220.11 g/mol
Appearance: Transparent oil

^{1}H NMR (400 MHz, $CDCl_3$) : 7.26ppm solvent ($CDCl_3$), 6.98ppm (m, 3H, **H**-Ph), 6.68ppm (dd, 1H, C**H**=CH_2), 6.5 (impurity DMAP), 5.68ppm (d, 1H, CH=CH**Htrans**), 5.24ppm (d, 1H, CH=C**Hcis**H), 3.83ppm (s, 3H, -OC**H₃**), 3ppm (impurity DMAP), 2.60ppm (impurity, butyric anhydride), 2.56ppm (t, 2H, OCOC$\mathbf{H_2}$$CH_2CH_3$), 2.40ppm (impurity, butyric acid), 1.79ppm (six, 2H, $OCOCH_2$C$\mathbf{H_2}$$CH_3$), 1.75ppm (impurity, butyric acid), 1.27ppm (impurity, butyric anhydride) 1.05ppm (t, 3H, $OCOCH_2CH_2$C$\mathbf{H_3}$), 0.95ppm (impurity, butyric acid).

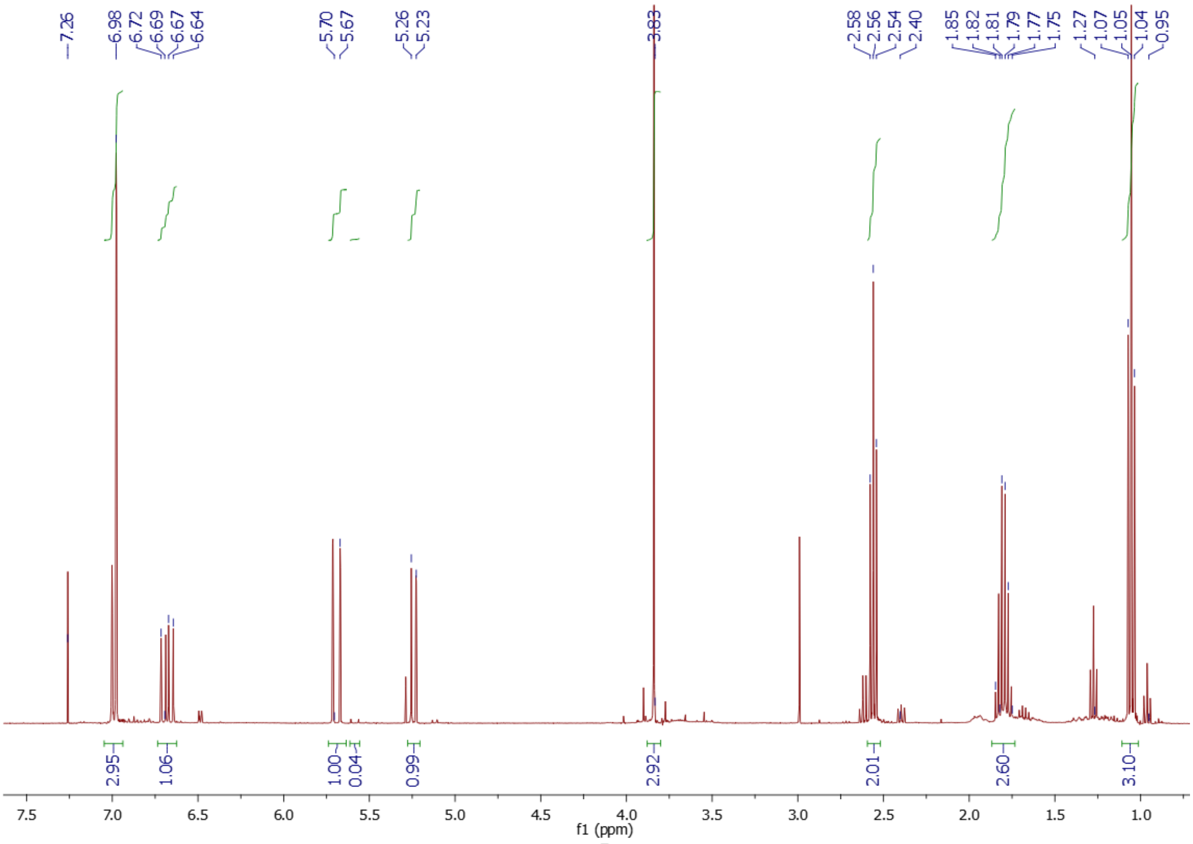

Figure S 7: *^{1}H NMR of But4VG in $CDCl_3$.*

GC-MS: presence of the pic (9.21 min) of MW= 220 g/mol representing But4VG, purity by GC-MS = 86%.
Presence of 4VG (7.18 min).

Sample Information

Sample Name	: ER017
Vial #	: 7
Injection Volume	: 1.00
Data File	: C:\GCMSsolution\Data\Project1\IAM\Elena RIGO\220622-ER017ok.qgd
Method File	: C:\GCMSsolution\Data\Project1\IAM\Elena RIGO\50-280 (split 30) début 2.5min + g
Tuning File	: C:\GCMSsolution\System\Tune1\2022.06.07.qgt

Chromatogram ER017 C:\GCMSsolution\Data\Project1\IAM\Elena RIGO\220622-ER017ok.qgd

Group #1

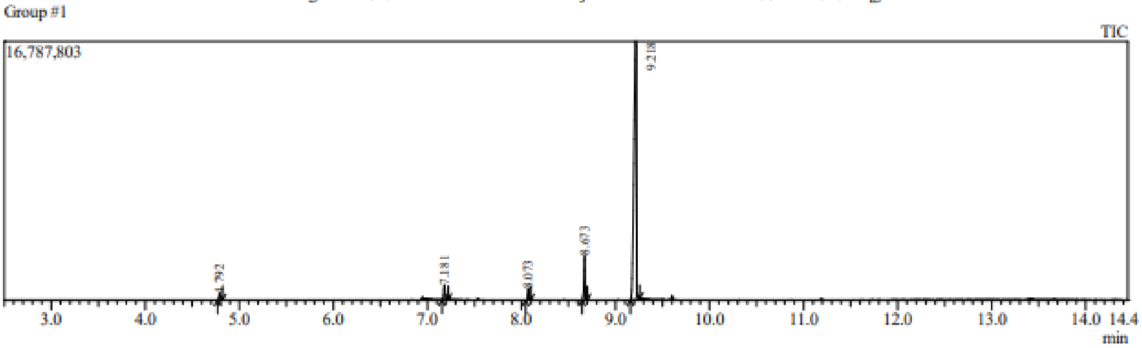

Peak Report

Peak#	R.Time	I.Time	F.Time	Area	Area%	Height	Name
1	4.792	4.770	4.823	405628	1.25	418896	DMAP
2	7.181	7.157	7.223	921248	2.84	974445	2-Methoxy-4-vinylphenol
3	8.073	8.047	8.100	635097	1.96	746247	?
4	8.673	8.647	8.703	2486142	7.66	2897468	DCC
5	9.218	9.160	9.260	28002988	86.29	16699942	But4VG (MW = 220g/mol)
				32451103	100.00	21736998	

Spectrum

Line#:1 R.Time:4.793(Scan#:689)

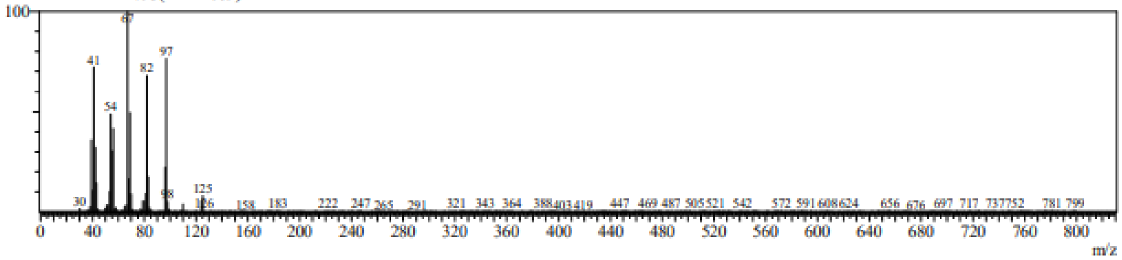

Line#:2 R.Time:7.180(Scan#:1405)

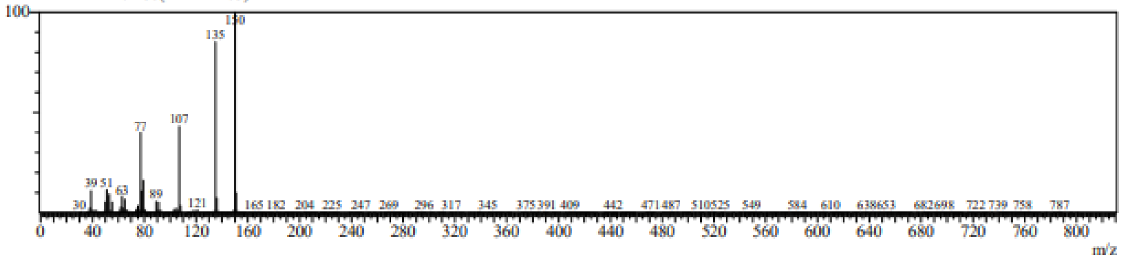

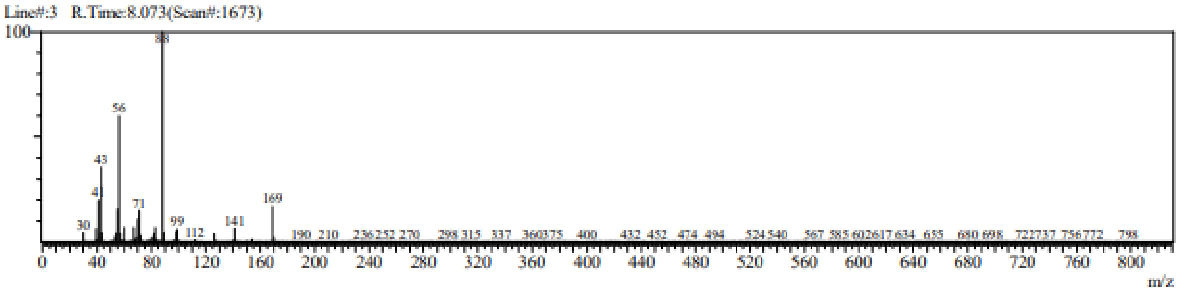

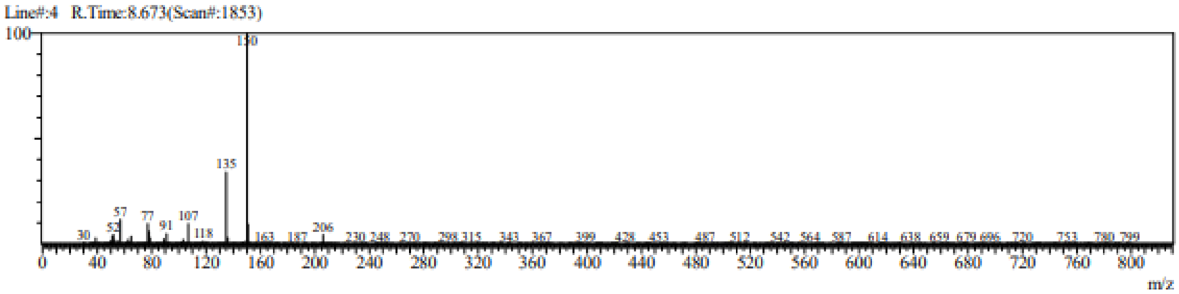

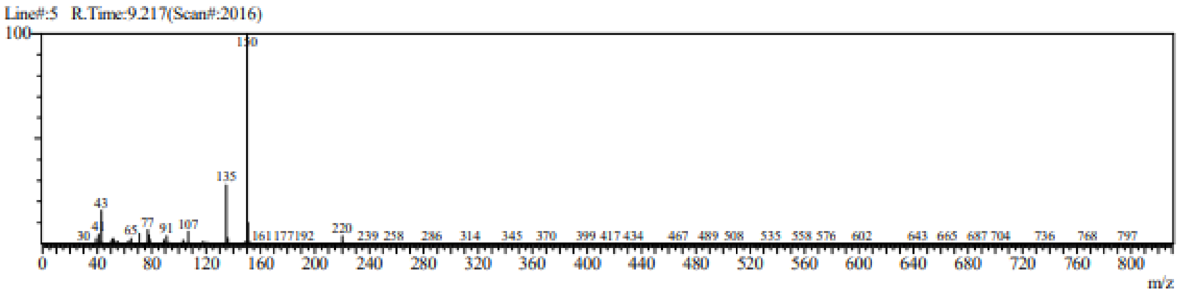

Figure S 8 : *GC chromatogram and MS spectrum of But4VG.*

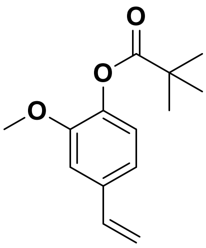

Acronym: Piv4VG
IUPAC Name: 2-methoxy-4-vinylphenyl pivalate
Chemical Formula: $C_{14}H_{18}O_3$
Molecular Weight: 234.13 g/mol
Appearance: Pale yellow oil
Melting point: -46°C

^{1}H NMR (400 MHz, $CDCl_3$) : 7.26ppm solvent ($CDCl_3$), 7.02ppm (m, 3H, **H**-Ph), 6.71ppm (dd, 1H, C**H**=CH_2), 5.72ppm (d, 1H, CH=CH**Htrans**), 5.26ppm (d, 1H, CH=C**Hcis**H), 4.2ppm (ethyl acetate, solvent), 3.85ppm (s, 3H, -OC$\mathbf{H_3}$), 2.08ppm (ethyl acetate), 1.39ppm (t, 3H, OCOC$\mathbf{H_3}$C$\mathbf{H_3}$C$\mathbf{H_3}$), 1.30ppm (impurity, pivalic anhydride), 1.28ppm (impurity, pivalic acid).

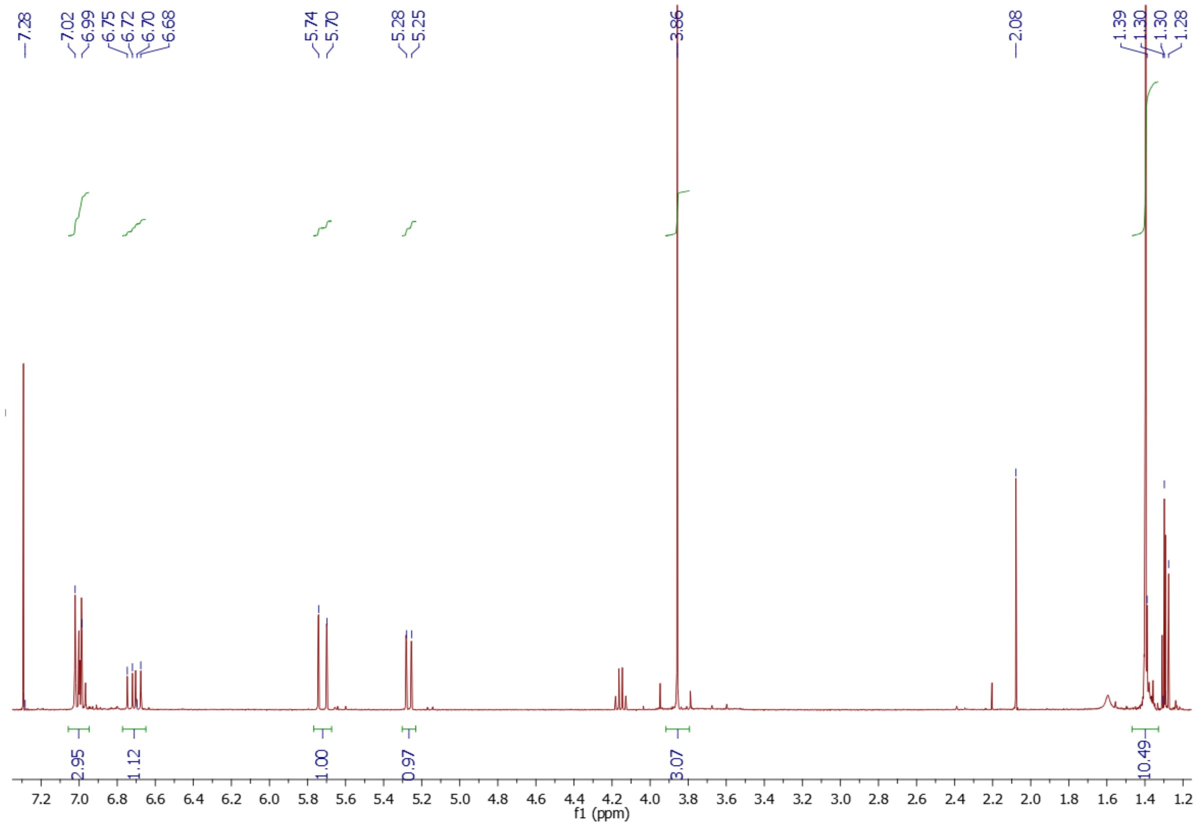

Figure S 9: *^{1}H NMR of Piv4VG in $CDCl_3$.*

GC-MS: presence of a pic (9.14 min) of MM= 234 g/mol representing Piv4VG, purity by GC-MS = 97%. Presence of 4VG (7.21 min).

Sample Information

Sample Name	: ER049
Vial #	: 6
Injection Volume	: 1.00
Data File	: C:\GCMSsolution\Data\Project1\IAM\Elena RIGO\220922-ER046 ok.qgd
Method File	: C:\GCMSsolution\Data\Project1\IAM\Elena RIGO\50-280 (split 30) début 2.5min + g
Tuning File	: C:\GCMSsolution\System\Tune1\2022.09.19.qgt

Chromatogram ER049 C:\GCMSsolution\Data\Project1\IAM\Elena RIGO\220922-ER046 ok.qgd

Group #1

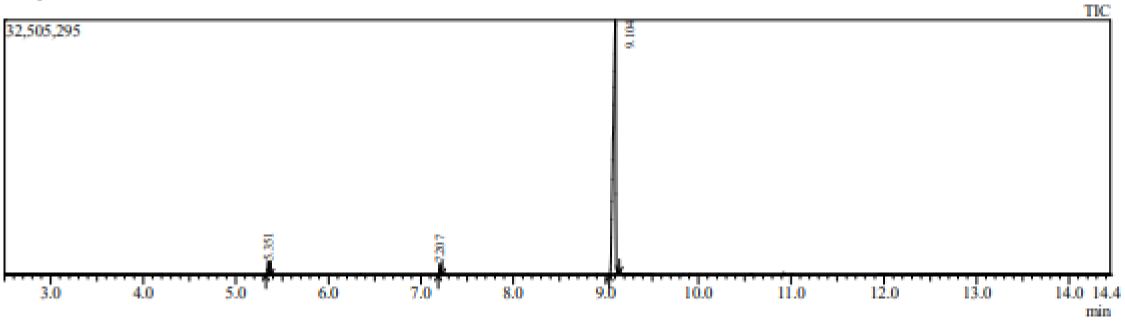

Peak Report

Peak#	R.Time	I.Time	F.Time	Area	Area%	Height	Name
1	5.351	5.327	5.377	1359124	2.00	1723007	2,2-Dimethylpropanoic anhydride
2	7.207	7.187	7.230	1199064	1.76	1384719	2-Methoxy-4-vinylphenol
3	9.104	9.037	9.147	65429804	96.24	32328895	Piv4VG (MW = 324 g/mol)
				67987992	100.00	35436621	

Spectrum

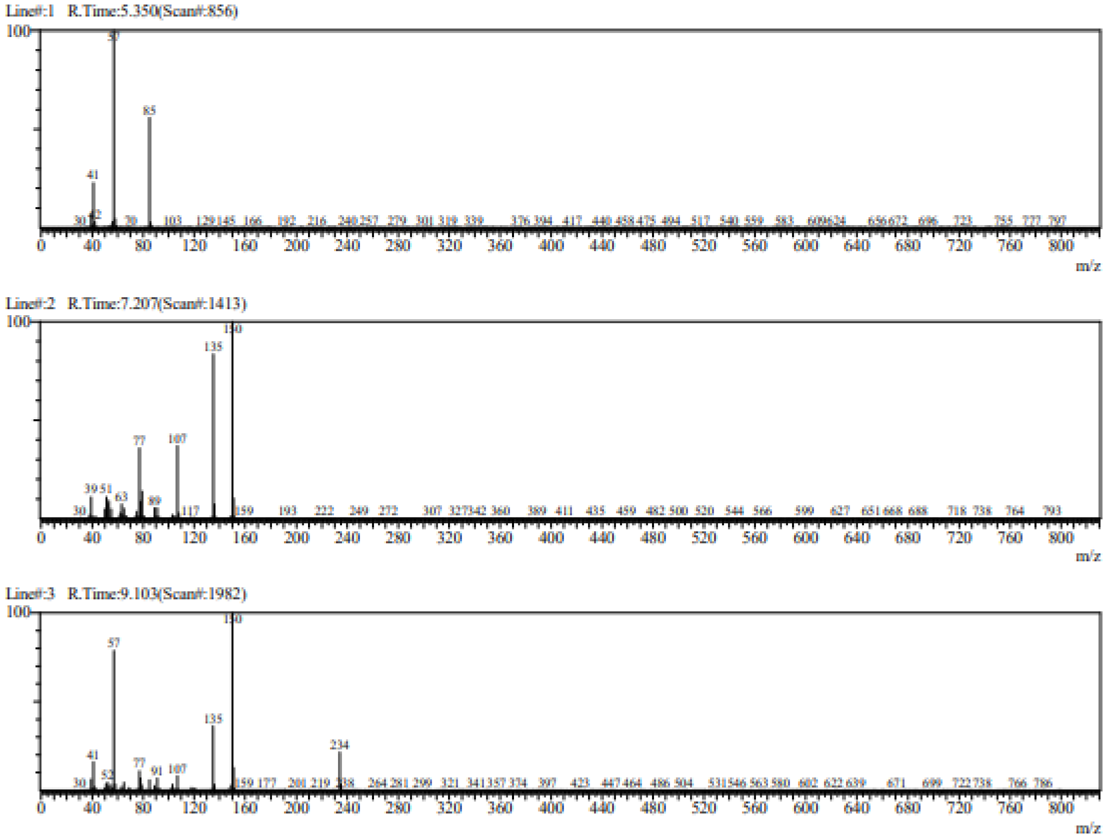

Figure S 10 : *GC chromatogram and MS spectrum of Piv4VG.*

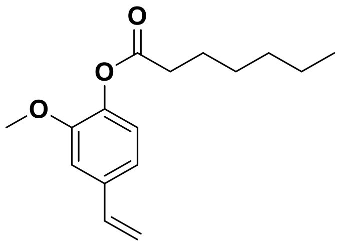

Acronym: Hept4VG
IUPAC Name: 2-methoxy-4-vinylphenyl heptanoate
Chemical Formula: $C_{16}H_{22}O_3$
Molecular Weight: 262.35 g/mol
Appearance: Transparent oil

^{1}H NMR (400 MHz, $CDCl_3$) of the product purified by chromatography column: 7.26ppm solvent ($CDCl_3$), 7ppm (m, 3H, **H**-Ph), 6.68pm (dd, 1H, C**H**=CH_2), 5.70ppm (d, 1H, CH=CH**Htrans**), 5.24ppm (d, 1H, CH=C**Hcis**H), 5.30 ppm (CH_2Cl_2, solvent), 3.84ppm (s, 3H, -OC**H**$_3$), 2.58ppm (t, 2H, OCOC**H**$_2$$CH_2$), 1,77 ppm (quint, 2H, OCOCH_2C**H**$_2$$CH_2$), , 1.44-1.36ppm (m, 6H), 0.93 ppm (t, 3H, CH_2C**H**$_3$).

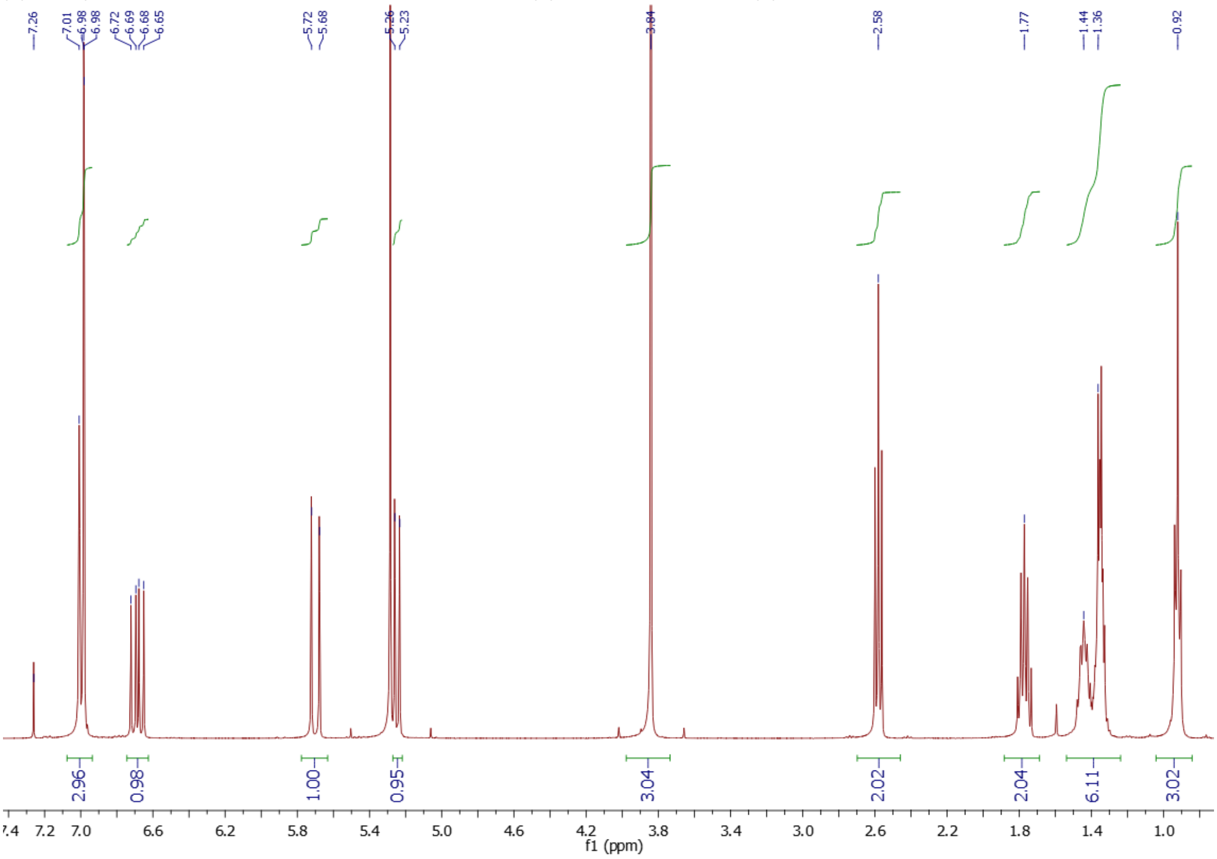

Figure S 11: *^{1}H NMR of Hept4VG in $CDCl_3$.*

GC-MS: presence of a pic (10.86 min) of MM= 262 g/mol representing Hept4VG, purity by GC-MS = 99%.
Presence of 4VG (10.26 min). The flow of carrier gas was 0.74mL/min for this analysis (instead of 1mL/min for the other GC analyses), explaining the longer retention time for 4VG.

Sample Information

Sample Name	: Hept4VG
Vial #	: 10
Injection Volume	: 1.00
Data File	: C:\GCMSsolution\Data\Project1\IAM\Elena RIGO\231205-Hept4VGok.qgd
Method File	: C:\GCMSsolution\Data\Project1\IAM\Elena RIGO\50-280 (split 30) début 2.5min + g
Tuning File	: C:\GCMSsolution\System\Tune1\2023.08.25.qgt

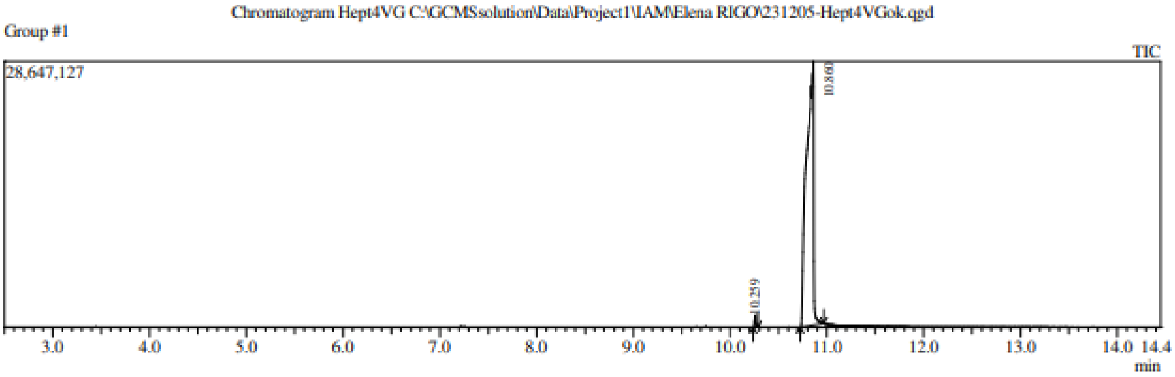

Peak Report

Peak#	R.Time	I.Time	F.Time	Area	Area%	Height	Name
1	10.259	10.240	10.287	1045819	0.71	1256158	4-vinyl guaiacol
2	10.860	10.723	10.970	145375711	99.29	27003418	Hept4VG
				146421530	100.00	28259576	

Spectrum

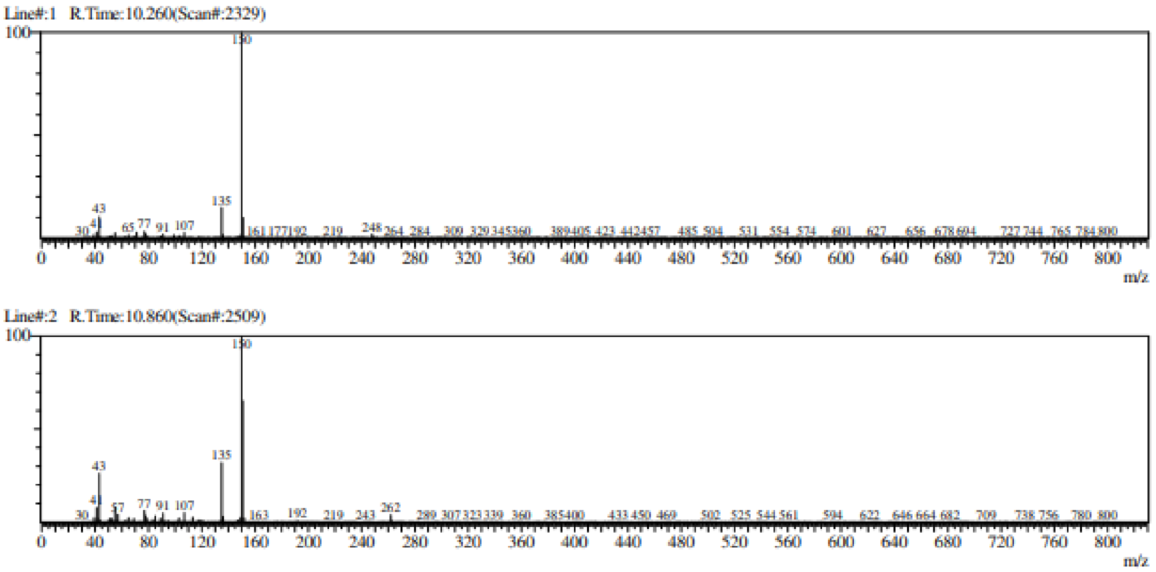

Figure S 12 : *GC chromatogram and MS spectrum of Hept4VG.*

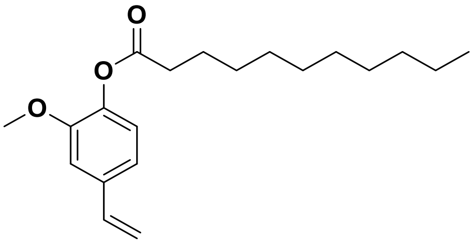

Acronym: Und4VG
IUPAC Name: 2-methoxy-4-vinylphenyl undecanoate
Chemical Formula: $C_{20}H_{30}O_3$
Molecular Weight: 318.22 g/mol
Appearance: Transparent oil

<u>^{1}H NMR (400 MHz, $CDCl_3$)</u> of the product purified by chromatography column: 7.26ppm solvent ($CDCl_3$), 7ppm (m, 3H, **H**-Ph), 6.69ppm (dd, 1H, C**H**=CH_2), 5.70ppm (d, 1H, CH=CH**Htrans**), 5.25ppm (d, 1H, CH=C**Hcis**H), 3.84ppm (s, 3H, -OC**H**$_3$), 2.59ppm (t, 2H, OCOC**H**$_2$$CH_2$), 1,78 ppm (quint, OCO$CH_2$C**H**$_2$$CH_2$), 1.45-1.30ppm (m, 14H), 0.91 ppm (t, 3H, CH_2C**H**$_3$).

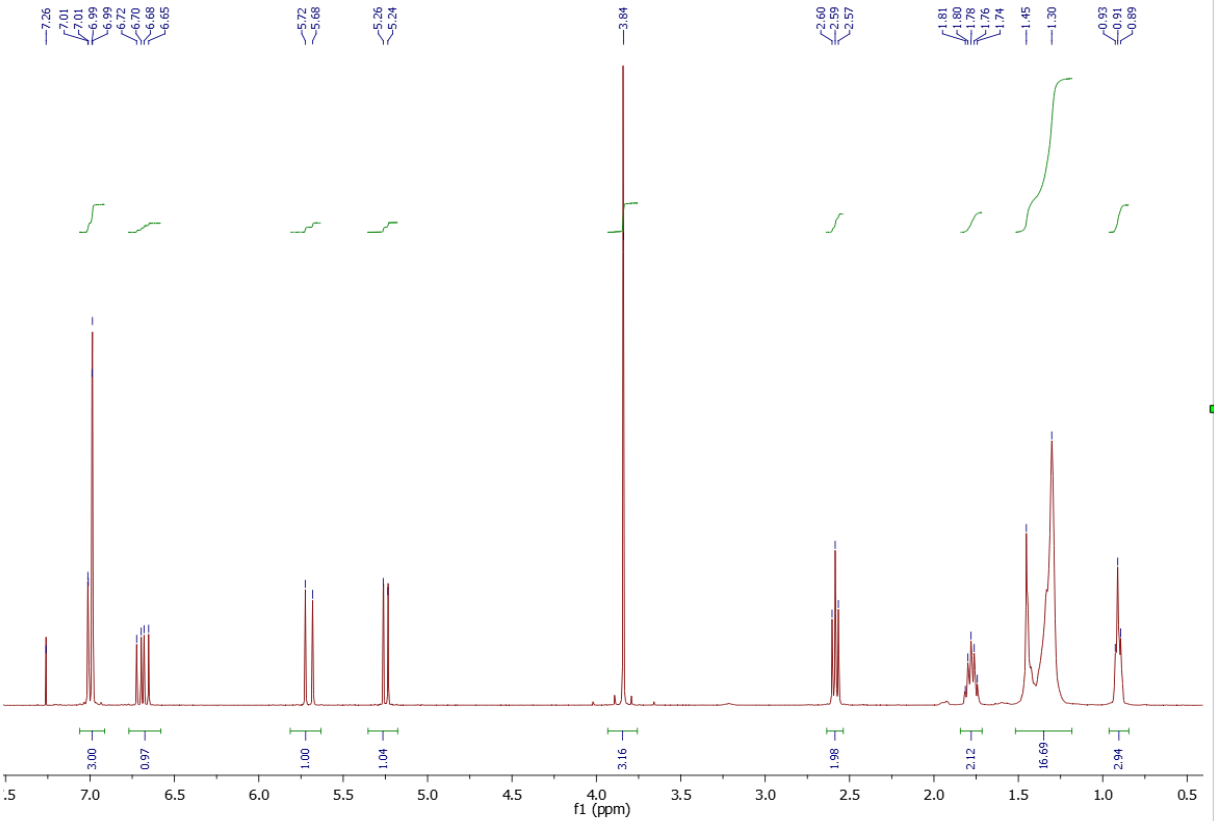

Figure S 13 : *^{1}H NMR of Und4VG in $CDCl_3$.*

<u>GC-MS</u>: presence of impurities
Signal at 9.028 min : 4VG
Signal at 11.63 min : DCC
Signal at 16.62 min: MW: 318 g/mol: Undecanoic4VG, purity by GC=98%.

Area Percent Report

Data Path : D:\Backup_ordi_syntho\GCMS\1\data\Elena\
Data File : ER132.D
Acq On : 27 Mar 2023 13:48
Operator : ER
Sample : 01er132
Misc :
ALS Vial : 20 Sample Multiplier: 1

Integration Parameters: autoint1.e
Integrator: ChemStation

Method : D:\MassHunter\GCMS\1\methods\generalmethod20230424.M
Title :

Signal : TIC: ER132.D\data.ms

peak #	R.T. min	first scan	max scan	last scan	PK TY	peak height	corr. area	corr. % max.	% of total
1	9.028	1031	1038	1048	BB	184807	2839098	0.67%	0.654%
2	11.635	1485	1494	1504	BB	417421	5817255	1.37%	1.339%
3	16.625	2336	2366	2416	BB	9868669	425664800	100.00%	98.007%

Sum of corrected areas: 434321152

generalmethod20230424.M Thu Apr 27 09:55:08 2023

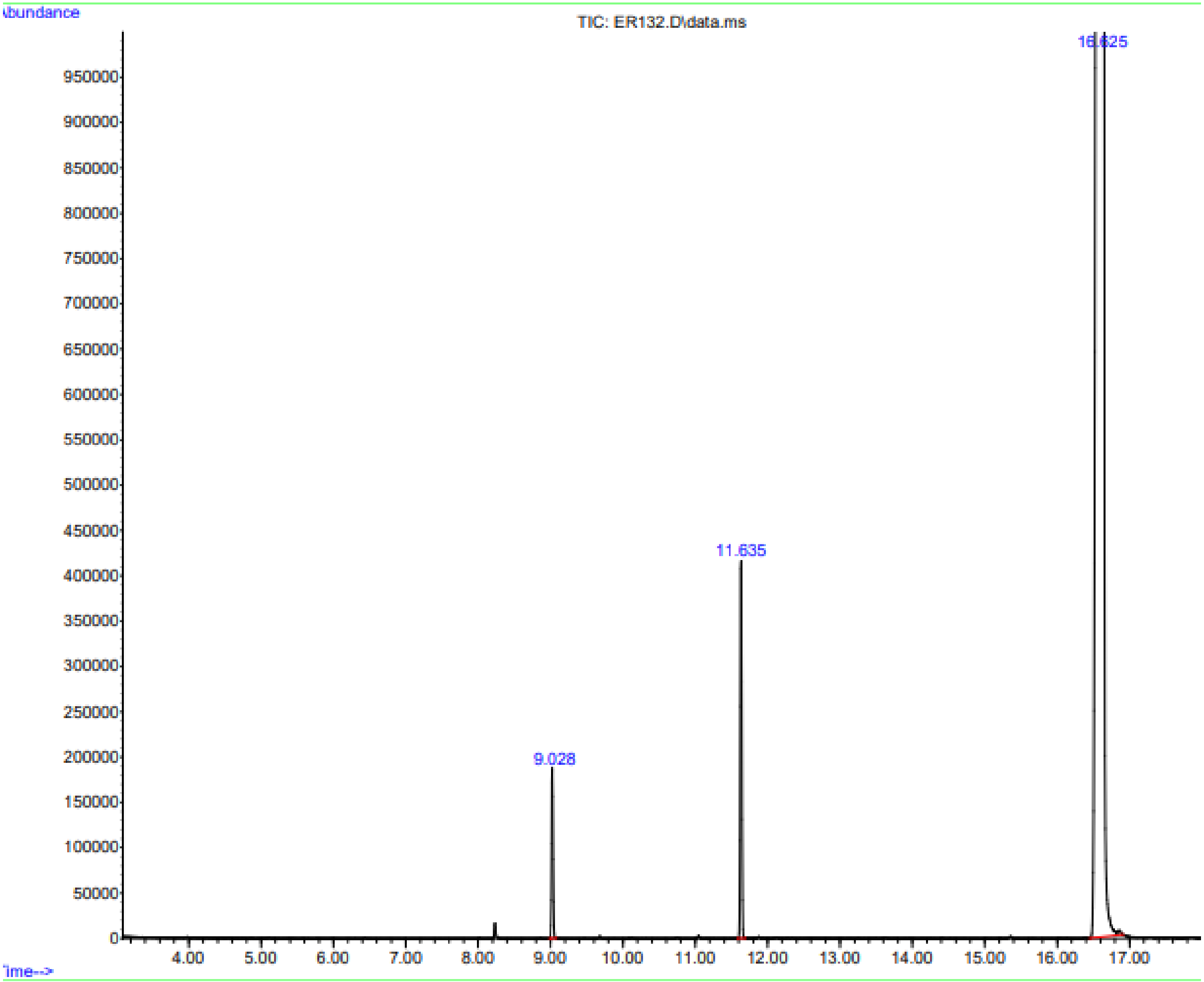

Impurity 9.028 min (4VG)

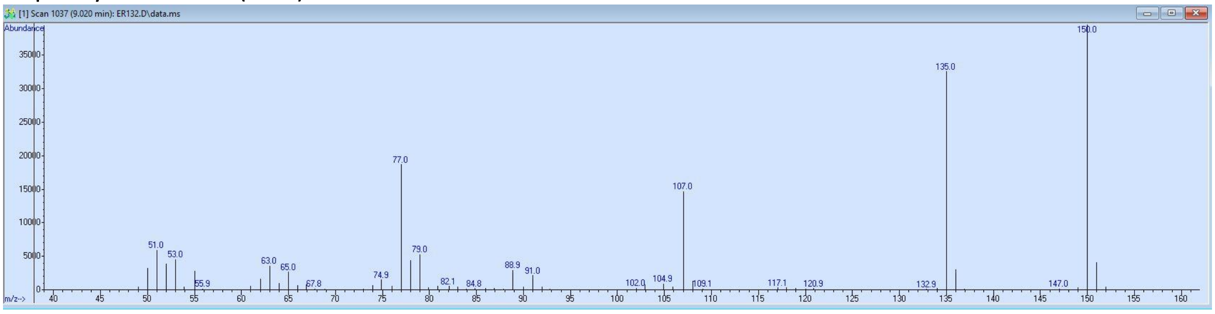

Impurity 11.635 min (DCC)

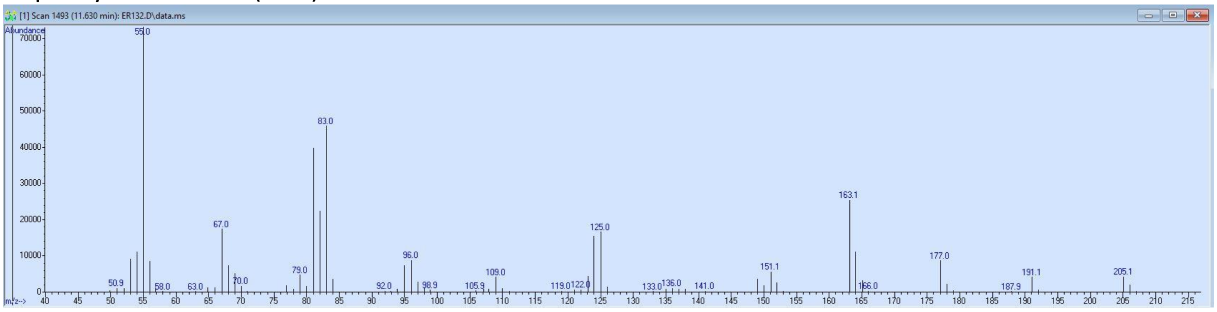

Und4VG 16.625 min

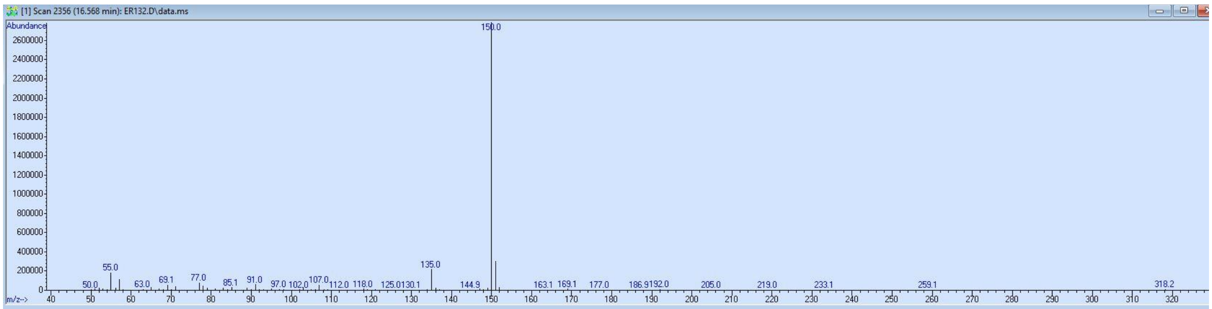

Figure S 14 *: GC chromatogram and MS spectrum of Und4VG.*

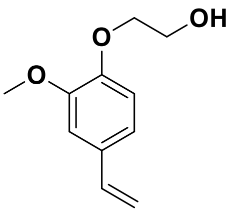

Acronym: HE4VG
IUPAC Name: 2-(2-methoxy-4-vinylphenol)ethan-1-ol
Chemical Formula: $C_{11}H_{14}O_3$
Molecular Weight: 194.09 g/mol
Appearance: White solid
Melting point: 61°C
^{1}H NMR (400 MHz, $CDCl_3$) : 7.26ppm solvent ($CDCl_3$), 6.92ppm (m, 3H, **H**-Ph), 6.64ppm (dd, 1H, C**H**=CH_2), 5.62ppm (d, 1H, CH=CH**Htrans**), 5.30 ppm (CH_2Cl_2, solvent), 5.16ppm (d, 1H, CH=C**Hcis**H), 4.11ppm (t, 2H, OC**H₂**...

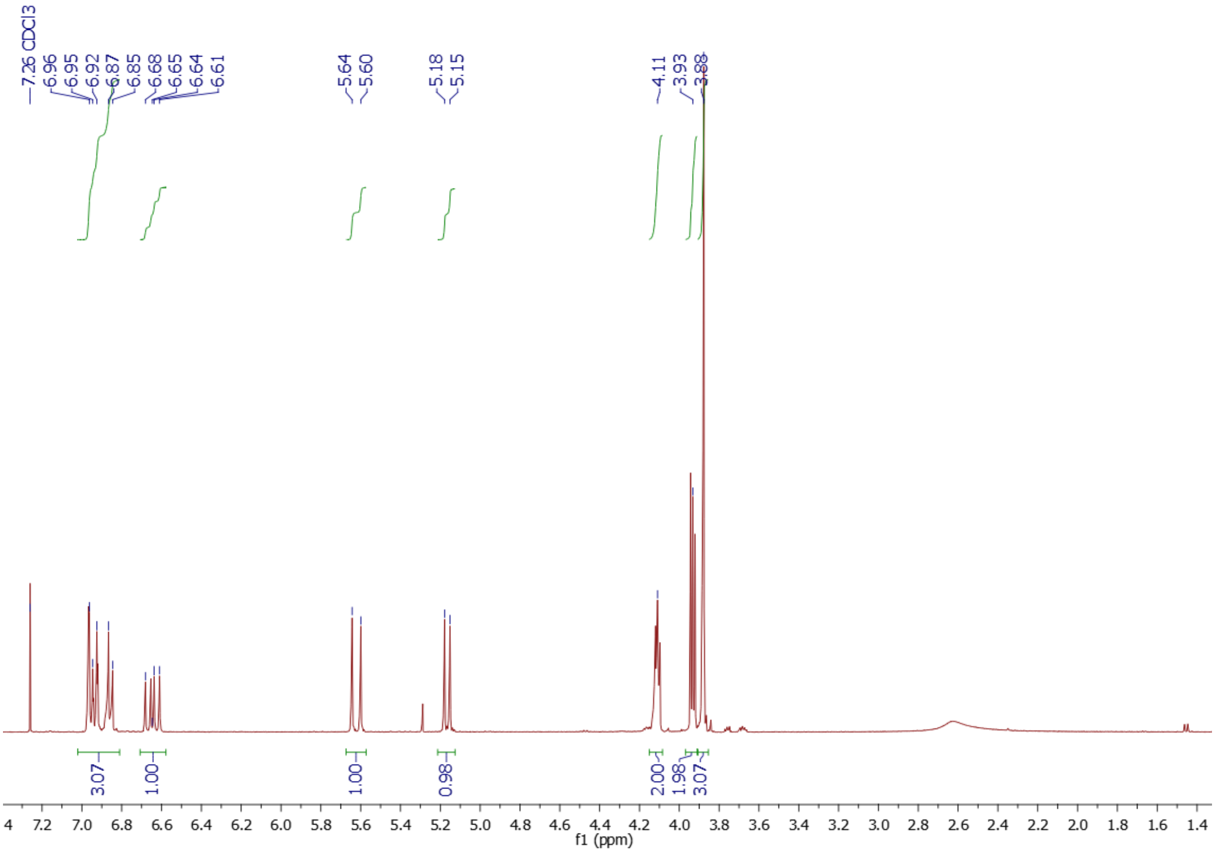

__Figure S 15 :__ ^{1}H NMR of HE4VG in $CDCl_3$.

<u>GC-MS</u>: presence of a pic (9.09 min) of MM= 194 g/mol representing HE4VG, purity by GC-MS = 87%. Presence of 4VG (7.21 min).
The signals at 12.5 min could be assigned to dimerization molecules.

Sample Information

Sample Name	: ER040
Vial #	: 21
Injection Volume	: 1.00
Data File	: C:\GCMSsolution\Data\Project1\IAM\Elena RIGO\220920-ER040ok.qgd
Method File	: C:\GCMSsolution\Data\Project1\IAM\Elena RIGO\50-280 (split 30) début 2.5min + g
Tuning File	: C:\GCMSsolution\System\Tune1\2022.09.19.qgt

Chromatogram ER040 C:\GCMSsolution\Data\Project1\IAM\Elena RIGO\220920-ER040ok.qgd

Group #1

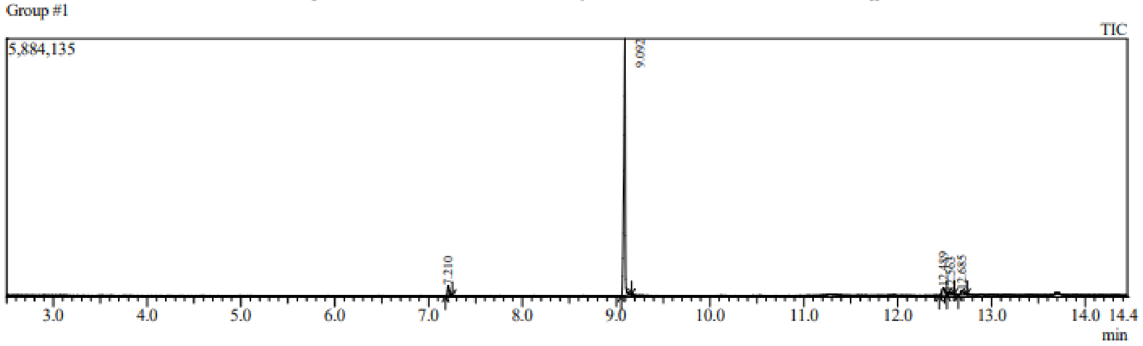

Peak Report

Peak#	R.Time	I.Time	F.Time	Area	Area%	Height	Name
1	7.210	7.180	7.257	234722	3.02	229384	2-Methoxy-4-vinylphenol
2	9.092	9.053	9.167	6834579	87.89	5845653	Hydroxyethyl4VG
3	12.489	12.443	12.527	379065	4.87	171714	Di-hydroxyethyl4VG (dimer)
4	12.563	12.527	12.603	128498	1.65	50565	Dimer-rearrangement
5	12.685	12.647	12.740	199128	2.56	99950	Dimer-rearrangement
				7775992	100.00	6397266	

Spectrum

Line#:1 R.Time:7.210(Scan#:1414)

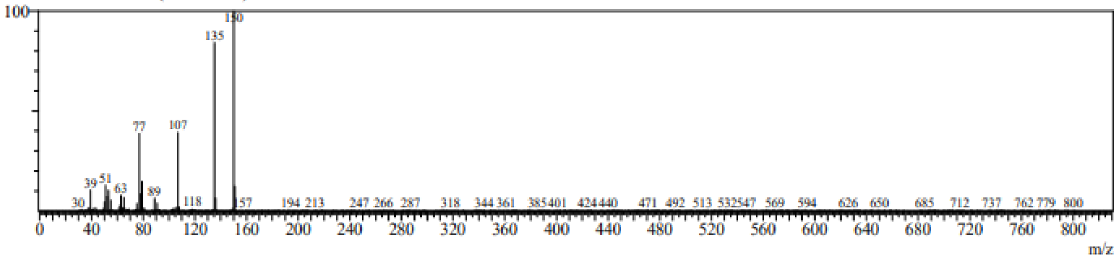

Line#:2 R.Time:9.093(Scan#:1979)

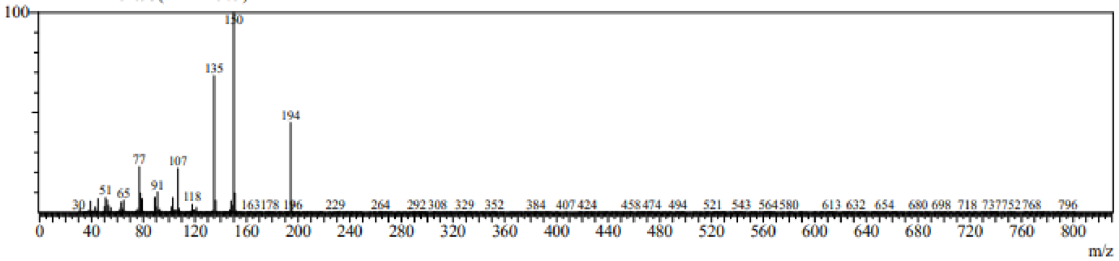

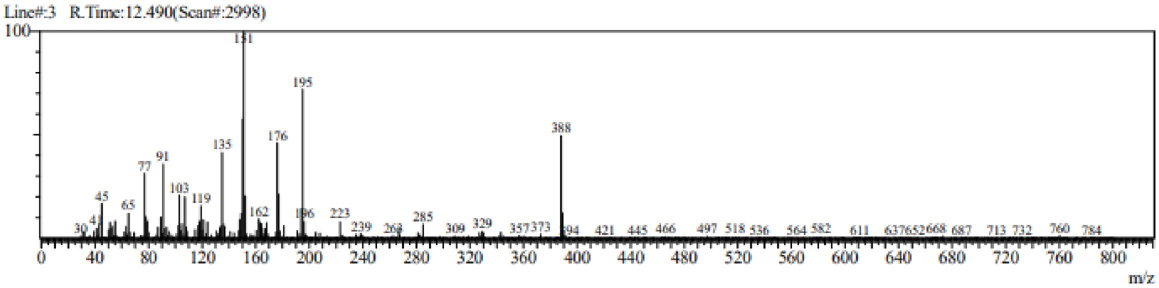

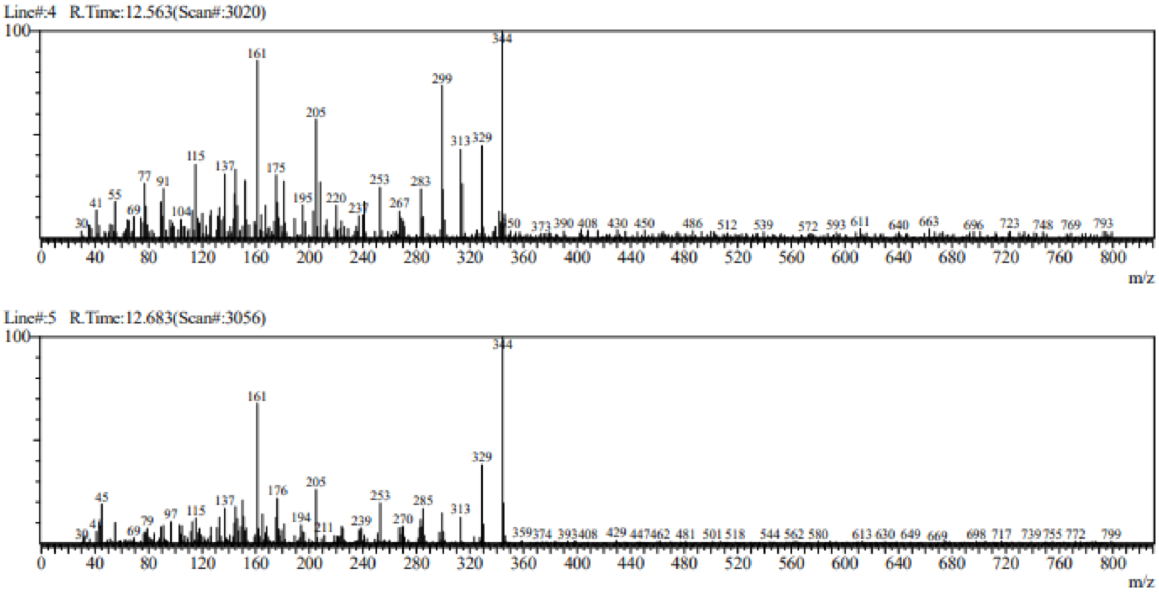

Figure S 16 : *GC chromatogram and MS spectrum of HE4VG.*

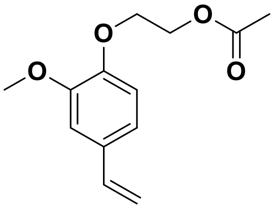

Acronym: AcHE4VG
IUPAC Name: 2-(2-methoxy-4-vinylphenoxy)ethyl acetate
Chemical Formula: $C_{13}H_{16}O_3$
Molecular Weight: 236.27 g/mol
Appearance: Pale yellow oil.

^{1}H NMR (400 MHz, $CDCl_3$) : 7.26ppm solvent ($CDCl_3$), 6.92ppm (m, 3H, **H**-Ph), 6.65ppm (dd, 1H, C**H**=CH_2), 5.63ppm (d, 1H, CH=CH**Htrans**), 5.16ppm (d, 1H, CH=C**Hcis**H), 4.44ppm (t, 2H, OC$\mathbf{H_2}$$CH_2$OC=O$CH_3$), 4.23ppm (t, 2H, O$CH_2C\mathbf{H_2}$O C=O$CH_3$), 4.17ppm (solvent, ethyl acetate), 3.89ppm (s, 3H, O-C$\mathbf{H_3}$), 2.22ppm (acetic anhydride, impurity), 2.10ppm (s, 3H, OC=OC$\mathbf{H_3}$), 2.04ppm and 1.25 ppm (solvent, ethyl acetate).

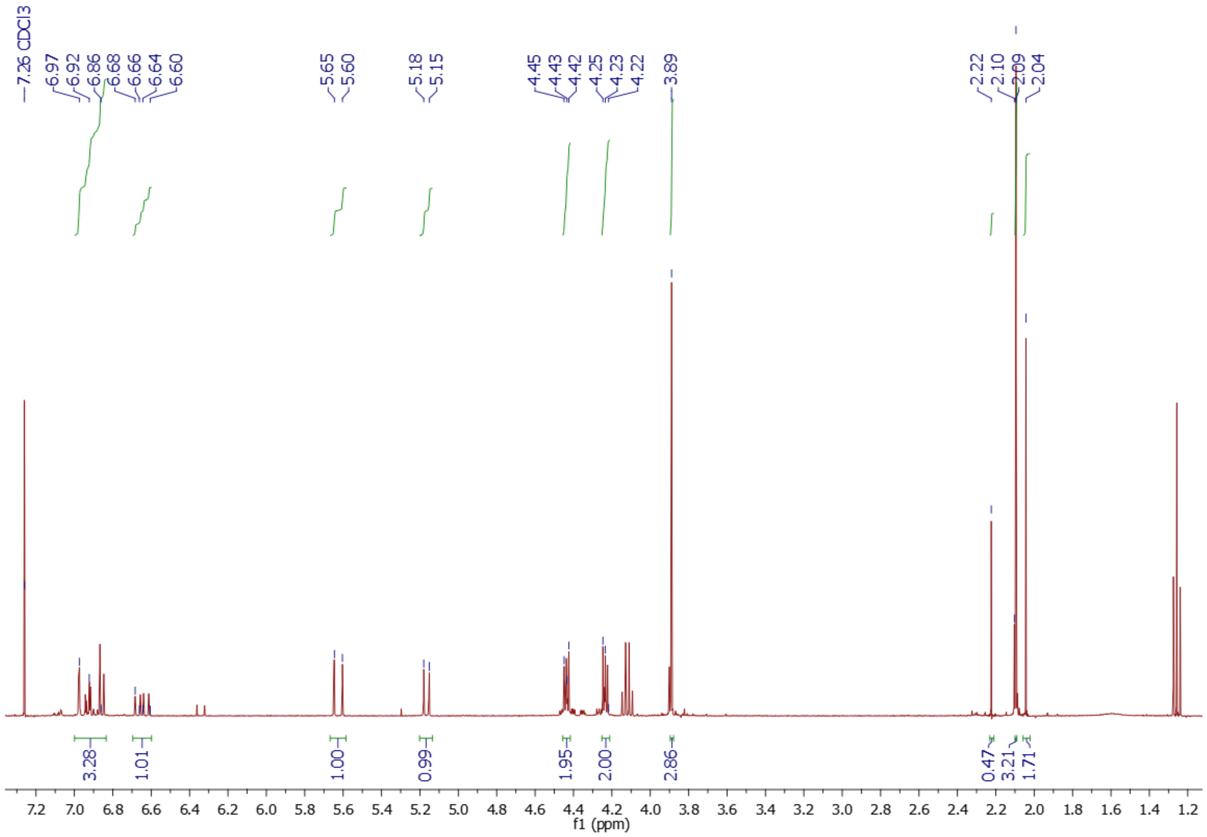

Figure S 17 : *^{1}H NMR of AcHE4VG in $CDCl_3$.*

<u>GC-MS</u>: presence of a pic (9.87 min) of MM= 236 g/mol representing AcHE4VG, purity by GC-MS = 95%. Presence of HE4VG (9.07 min).

Sample Information

Sample Name	: ER036
Vial #	: 19
Injection Volume	: 1.00
Data File	: C:\GCMSsolution\Data\Project1\IAM\Elena RIGO\220920-ER036ok.qgd
Method File	: C:\GCMSsolution\Data\Project1\IAM\Elena RIGO\50-280 (split 30) début 2.5min + gs
Tuning File	: C:\GCMSsolution\System\Tune1\2022.09.19.qgt

Chromatogram ER036 C:\GCMSsolution\Data\Project1\IAM\Elena RIGO\220920-ER036ok.qgd

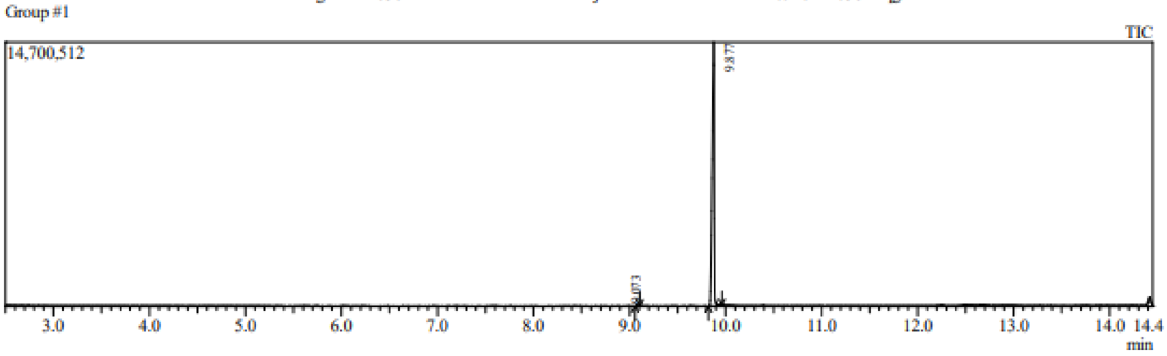

Peak Report

Peak#	R.Time	I.Time	F.Time	Area	Area%	Height	Name
1	9.073	9.057	9.107	78102	0.39	82777	HE4VG
2	9.877	9.823	9.963	20121722	99.61	14655129	HEAc4VG (MW = 236 g/mol)
				20199824	100.00	14737906	

Spectrum

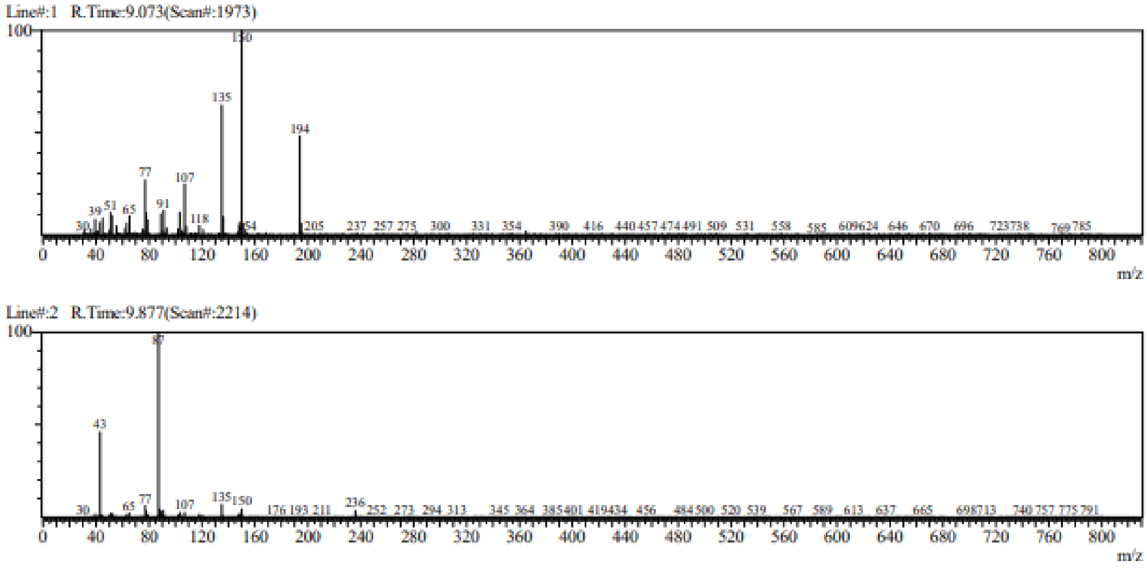

Figure S 18 : *GC chromatogram and MS spectrum of AcHE4VG.*

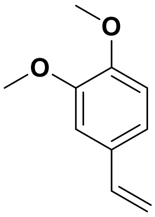

Acronym: Met4VG
IUPAC Name: 1,2-dimethoxy-4-vinylbenzene
Chemical Formula: $C_{10}H_{12}O_2$
Molecular Weight: 164.08 g/mol
Appearance: Yellow liquid
Melting point: 3°C

^{1}H NMR (400 MHz, $CDCl_3$) : 7.26ppm solvent ($CDCl_3$), 6.98-6.84ppm (m, 3H, **H**-Ph), 6.65ppm (dd, 1H, C**H**=CH_2), 5.61ppm (d, 1H, CH=CH**Htrans**), 5.15ppm (d, 1H, CH=C**Hcis**H), 3.91ppm (s, 3H, OC**H$_3$**), 3.88 ppm (s, 3H, OC**H$_3$**), 2.17ppm (acetone solvent), 1.57ppm (impurity, water).

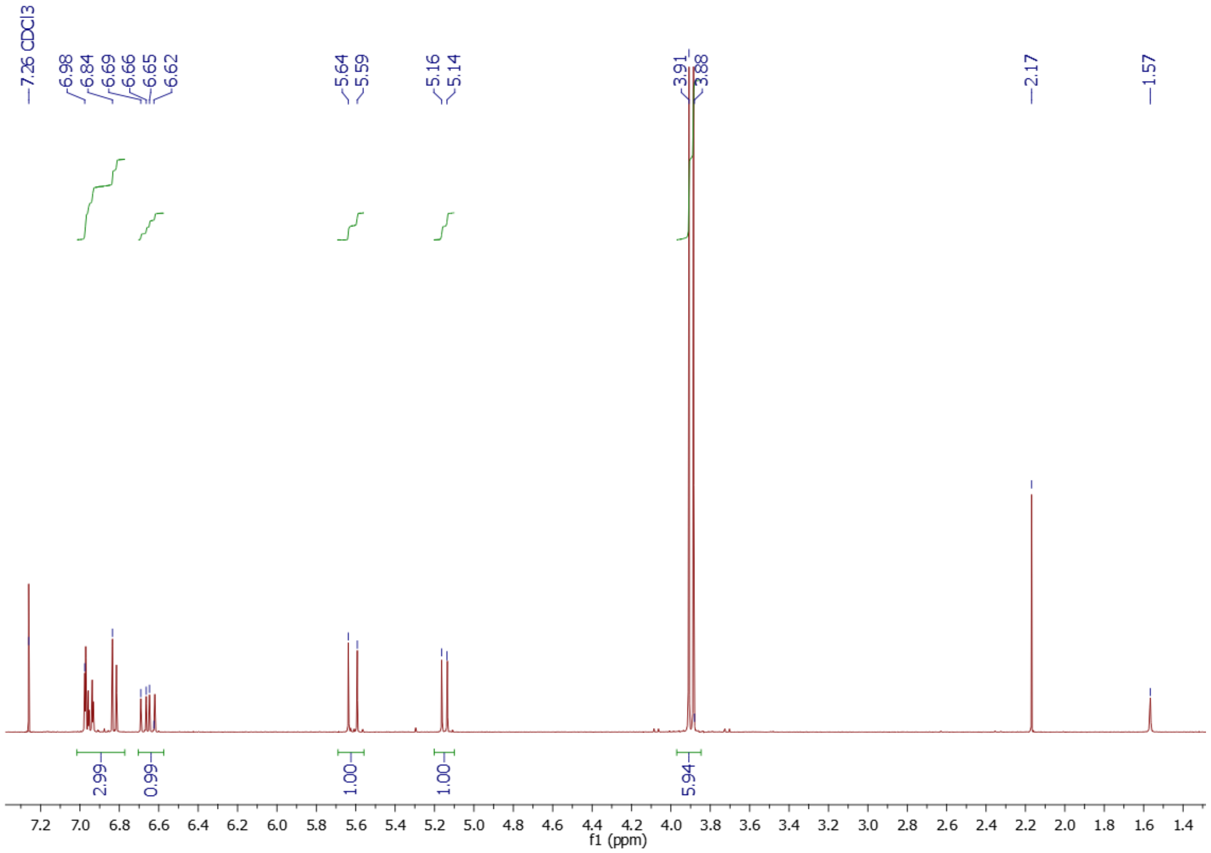

Figure S 19: *^{1}H NMR of Met4VG in $CDCl_3$.*

GC-MS: presence of a pic (7.56 min) of MM= 164 g/mol representing Met4VG, purity by GC-MS = 97%. Presence of 4VG (7.21 min).

Sample Information

Sample Name	:
Vial #	: 17
Injection Volume	: 1.00
Data File	: C:\GCMSsolution\Data\Project1\IAM\Elena RIGO\221013-ER064 ok.qgd
Method File	: C:\GCMSsolution\Data\Project1\IAM\Elena RIGO\50-280 (split 30) début 2.5min + g
Tuning File	: C:\GCMSsolution\System\Tune1\2022.09.19.qgt

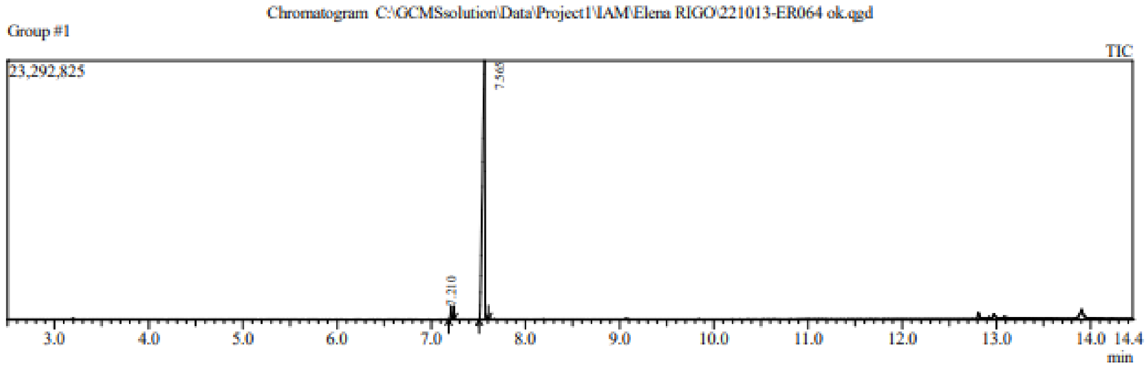

Peak Report

Peak#	R.Time	I.Time	F.Time	Area	Area%	Height	Name
1	7.210	7.183	7.243	1120057	2.52	1225463	2-Methoxy-4-vinylphenol
2	7.565	7.507	7.607	43370421	97.48	23237883	Met4VG (MW = 164 g/mol)
				44490478	100.00	24463346	

Spectrum

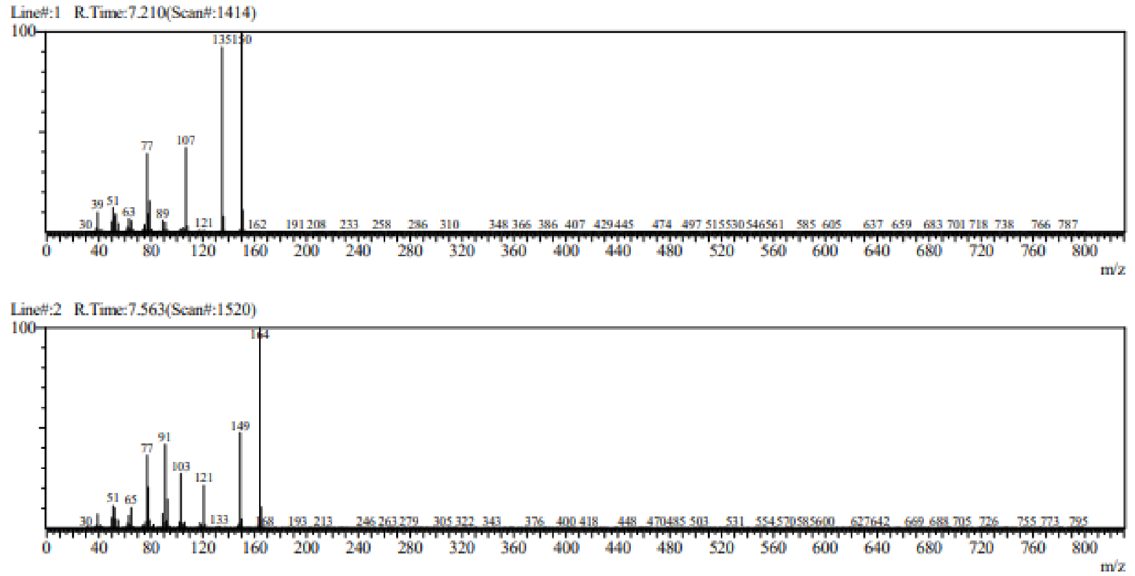

Figure S 20 : *GC chromatogram and MS spectrum of Met4VG.*

Calculation of green metrics

Table S 1 : *Details of green metrics calculations.*

**Ac4VG**	4VG	Acetic Anhydride	AcONa	Ac4VG	*Yield* (%)	*AE* (%)	*E-factor* (kg waste/kg product)	*C-efficiency* (%)	*RME* (%)	*Biobased carbon* (%)
MW (g/mol)	150.17	102.09	82.03	192.08						
nb of C	9	4	2	11						
m (g)	1.8	1.5	0.041							
n (mol)	0.012	0.015	0.0005							
m exp (g)				2.190						
n exp (mol)				0.011						
					95	76	0.5	74	66	82

**Prop4VG Route 1**	4VG	Propionic Anhydride	PropONa	Prop4VG	*Yield* (%)	*AE* (%)	*E-factor* (kg waste/kg product)	*C-efficiency* (%)	*RME* (%)	Biobased carbon (%)
MW (g/mol)	150.17	130.142	96.07	206						
nb of C	9	6	3	12						
m (g)	1.8	1.56	0.48							
n (mol)	0.0120	0.0120	0.0050							
m exp (g)				1.333						
n exp (mol)				0.006						
					54	73	1.9	40	35	75

**Prop4VG Route 2**	4VG	Propionic acid	DCC	DMAP	CH_2Cl_2	Prop4VG	*Yield* (%)	*AE* (%)	*E-factor* (kg waste/kg product)	*C-efficiency* (%)	*RME* (%)	*Biobased carbon* (%)
MW (g/mol)	150.17	76.08	206.33	122.17		206						
nb of C	9	3	13	7		12						
m (g)	1.8	0.98	2.7	0.36	38							
n (mol)	0.012	0.013	0.013	0.003								
m exp (g)						2.099						
n exp (mol)						0.010						
							85	91	19.9	36	36	100

**But4VG Route 1**	4VG	Butyric Anhydride	ButONa	But4VG	*Yield* (%)	*AE* (%)	*E-factor* (kg waste/kg product)	*C-efficiency* (%)	*RME* (%)	*Biobased carbon* (%)
MW (g/mol)	150.17	158.19	110.09	220						
nb of C	9	8	4	13						
m (g)	5	5.27	0.37							
n (mol)	0.033	0.033	0.003							
m exp (g)				6.007						
n exp (mol)				0.027						
					82	71	0.8	61	56	69

**But4VG Route 2**	4VG	Butyric acid	DCC	DMAP	CH_2Cl_2	But4VG	*Yield* (%)	*AE* (%)	*E-factor* (kg waste/kg product)	*C-efficiency* (%)	*RME* (%)	*Biobased carbon* (%)
MW (g/mol)	150.17	88.11	206.33	122.17		220						
nb of C	9	4	13	7		13						
m (g)	1.8	1.04	2.7	0.36	38							
n (mol)	0.012	0.012	0.013	0.003								
m exp (g)						2.611						
n exp (mol)						0.012						
							99	92	15.8	45	44	100

**Piv4VG Route 1**	4VG	Pivalic Anhydride	PivONa	But4VG	*Yield* (%)	*AE* (%)	*E-factor* (kg waste/kg product)	*C-efficiency* (%)	*RME* (%)	*Biobased carbon* (%)
MW (g/mol)	150.17	186.25	124.11	231						
nb of C	9	8	5	14						
m (g)	1.5	1.86	0.12							
n (mol)	0.010	0.010	0.001							
m exp (g)				1.569						
n exp (mol)				0.007						
					68	69	1.2	54	45	64

**Piv4VG Route 2**	4VG	Pivalic acid	DCC	DMAP	CH_2Cl_2	Piv4VG	*Yield* (%)	*AE* (%)	*E-factor* (kg waste/kg product)	*C-efficiency* (%)	*RME* (%)	*Biobased carbon* (%)

MW (g/mol)	150.17	102.13	206.33	122.17		231						
nb of C	9	5	13	7		14						
m (g)	1.8	1.21	2.7	0.36	38							
n (mol)	0.012	0.012	0.013	0.003								
m exp (g)						1.412						
n exp (mol)						0.006						
							51	92	30.2	24	23	64

**Hept4VG**	4VG	Heptanoic acid	DCC	DMAP	CH_2Cl_2	Hept4VG	*Yield* (%)	*AE* (%)	*E-factor* (kg waste/kg product)	*C-efficiency* (%)	*RME* (%)	*Biobased carbon* (%)
MW (g/mol)	150.17	130.18	206.33	122.17		262						
nb of C	9	7	13	7		16						
m (g)	10	10.4	15.11	0.81	211							
n (mol)	0.067	0.080	0.073	0.007								
m exp (g)						13.260						
n exp (mol)						0.051						
							76	93	17.6	38	37	100

**Und4VG**	4VG	Undecanoic acid	DCC	DMAP	CH_2Cl_2	Und4VG	*Yield* (%)	*AE* (%)	*E-factor* (kg waste/kg product)	*C-efficiency* (%)	*RME* (%)	*Biobased carbon* (%)
MW (g/mol)	150.17	186.29	206.33	122.17		318						
nb of C	9	11	13	7		20						
m (g)	5	6.2	7.55	0.4	110							

n (mol)	0.033	0.033	0.037	0.003								
m exp (g)						10.376						
n exp (mol)						0.033						
							98	95	11.4	56	54	100

**HE4VG**	4VG	Ethylene carbonate	DBN (cat)	HE4VG	*Yield* (%)	*AE* (%)	*E-factor* (kg waste/kg product)	*C-efficiency* (%)	*RME* (%)	*Biobased carbon* (%)
MW (g/mol)	150.17	88.02	124.1	194.09						
nb of C	**9**	3	7	11						
m (g)	1.5	0.97	0.62							
n (mol)	0.010	0.011	0.005							
m exp (g)				1.415						
n exp (mol)				0.007						
					73	81	1.2	51	46	82

**AcHE4VG**	HE4VG	Acetic Anhydride	AcONa	HEAc4VG	*Yield* (%)	*AE* (%)	*E-factor* (kg waste/kg product)	*C-efficiency* (%)	*RME* (%)	*Biobased carbon* (%)
MW (g/mol)	194.09	102.09	82.03	236.27						
nb of C	**11**	4	2	13						
m (g)	0.37	0.24	0.0075							
n (mol)	0.002	0.002	0.0001							
m exp (g)				0.158						
n exp (mol)				0.001						

					35	80	2.9	28	26	69

**Met4VG**	4VG	Iodiomethane	Potassium carbonate	Acetone	Met4VG	*Yield* (%)	*AE* (%)	*E-factor* (kg waste/kg product)	*C-efficiency* (%)	*RME* (%)	*Biobased carbon* (%)
MW (g/mol)	150.17	141.94	138.21		164.08						
nb of C	9	1	1		10						
m (g)	1.5	14.7	6.8	50							
n (mol)	0.010	0.104	0.049								
m exp (g)					1.606						
n exp (mol)					0.010						
						98	56	44.4	40	10	90

Results of homopolymerizations

Degradative chain transfer to 4VG:

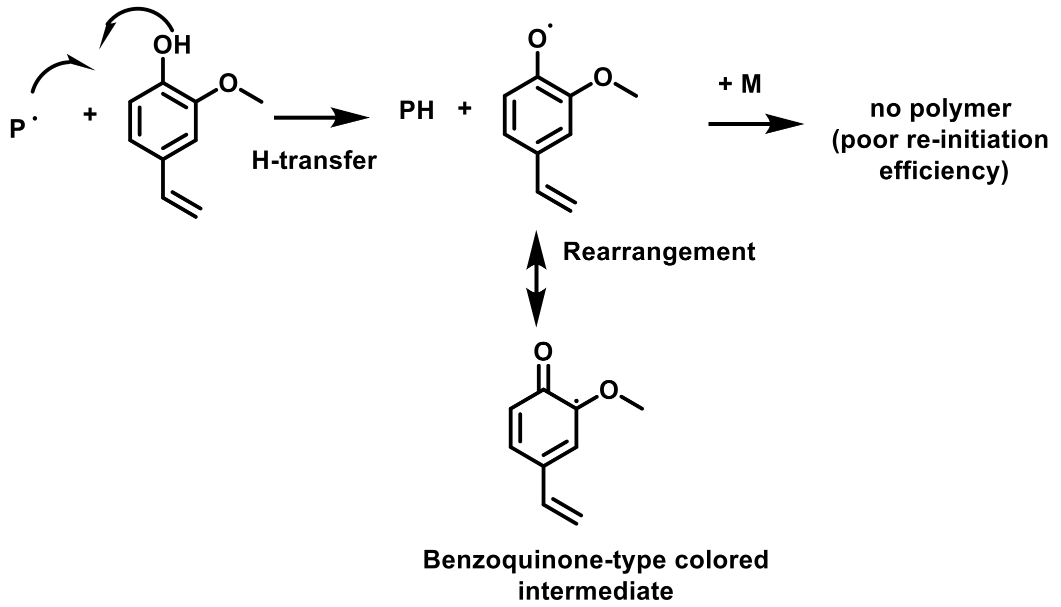

Figure S21: *Hypothetical mechanism of degradative chain transfer to 4VG, which can cause its inhibitor power.*

SEC of poly(Ac4VG) homopolymers produced in the presence of 4VG:

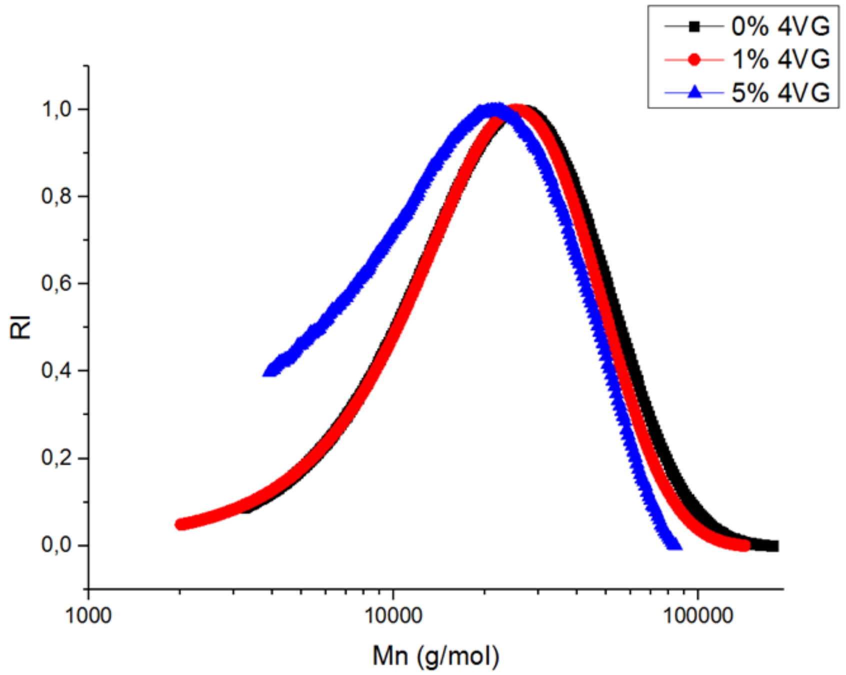

Figure S22: *SEC curves of poly(Ac4VG) homopolymers produced in the presence of quantified amount of 4VG (0, 1, and 5 wt% based on monomers). SEC calibration was performed with PMMA narrow standards.*

Kinetics of polymerization of 4VG derivatives:

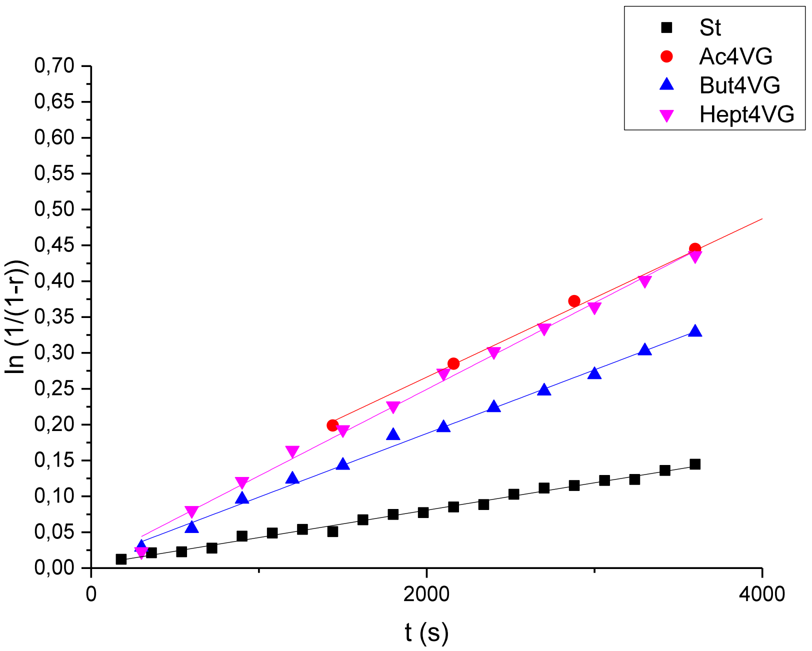

Figure S23: *Evolution of ln([M]$_0$/[M]) versus time for solution homopolymerization of styrene and 4VG-derivatives in deuterated-toluene, initiated by AIBN at 70°C (r is the fractional conversion of monomer) monitored by online ^{1}H-NMR spectroscopy.*

The measurements uncertainties (integral value I) by ^{1}H-NMR are typically less than $\Delta I/I$ = 1%[1]. The monomer fractional conversion r is given by $r = [1 - (I_{monomer,t}/I_{InternalStandard,t})/(I_{monomer,0}/I_{InternalStandard,0})]$. Thus, $\Delta r/r = \Delta I_{monomer,t}/I_{monomer,t} + \Delta I_{InternalStandard,t}/I_{InternalStandard,t} + \Delta I_{monomer,0}/I_{monomer,0} + \Delta I_{InternalStandard,0}/I_{InternalStandard,0} = 4 \times \Delta I/I$ = $4 \times 1\% = 4\%$. Therefore, the relative error uncertainty on the monomer conversion is less than $\Delta r/r = 4\%$.

a) Monomers integration and conversion at each time for homopolymerizations in toluene at 70°C initiated by AIBN, as reported in **Figure 7**.

Table S2: *Experimental data for monomer conversion at each time, reported in* ***Figure*** 7.

t (h)	Integral IS[a]	Integral Ac4VG	Conversion Ac4VG (%)	Integral 4VG	Conversion 4VG (%)	Integral Prop4VG	Conversion Prop4VG (%)	Integral But4VG	Conversion But4VG (%)	Integral Piv4VG	Conversion Piv4VG (%)	Integral St	Conversion St (%)	Integral HE4VG	Conversion HE4VG (%)	Integral Met4VG	Conversion Met4VG (%)	Integral AcHE4VG	Conversion AcHE4VG (%)	Integral Und4VG	Conversion Und4VG (%)	Integral Hept4VG	Conversion Hept4VG (%)
**0**	**1**	0.815	0	0.90	0	0.885	0	1.05	0	0.885	0	4.425	0	0.865	0	0.9	0	0.78	0	0.735	0	0.96	0
**0.5**	**1**	n.m .	n.m .	0.88	2	0.87	2	n.m.	n.m.	0.84	5	4.22	5	n.m.	n.m.	0.89	1	0.74	5	n.m.	n.m.	0.96	11
**1**	**1**	0.705	13	0.87	3	0.815	8	0.635	40	0.825	7	4.11	7	0.815	6	0.845	6	0.685	12	0.715	3	0.96	14
**1.5**	**1**	0.67	18	0.86	4	0.8	10	n.m.	n.m.	0.805	9	n.m .	n.m .	0.82	5	n.m.	n.m.	n.m.	n.m.	n.m.	n.m.	n.m .	n.m .
**2**	**1**	0.54	34	0.86	4	0.785	11	0.435	59	n.m .	n.m .	3.96	11	0.82	5	0.77	14	0.56	28	0.64	13	0.96	21
**3**	**1**	0.545	33	0.84	7	0.68	23	0.395	62	n.m .	n.m .	3.59	19	0.82	5	0.75	17	0.53	32	0.53	28	0.96	33
**4**	**1**	0.515	37	n.m.	n.m.	0.635	28	0.315	70	0.61	31	3.4	23	0.7	19	0.69	23	0.45	42	0.56	24	0.96	47
**5**	**1**	0.46	44	0.82	9	0.61	31	0.29	72	0.56	37	3.29	26	0.675	22	0.665	26	0.4	49	0.4	46	0.95	54
**6**	**1**	0.415	49	n.m .	n.m .	0.56	37	0.24	77	0.495	44	2.87	35	n.m.	n.m.	0.615	32	0.36	54	0.37	50	0.95	60
**7**	**1**	0.365	55	n.m .	n.m .	0.54	39	0.215	80	0.49	45	2.775	37	0.63	27	0.57	37	0.34	56	n.m.	n.m.	0.95	63
**8**	**1**	0.3	63	0.82	9	0.52	41	0.18	83	0.48	46	2.62	41	0.59	32	0.54	40	0.29	63	0.31	58	0.95	67
**24**	**1**	0.095	88	0.71	21	0.125	86	0.05	95	0.165	81	0.15	97	0.14	84	0.22	76	0.04	95	0.14	81	0.95	88
**48**	**1**			0.59	34																		

[a]The integration of the IS had a value of 1.

b) Experimental conditions for solution homopolymerizations in toluene at 70°C initiated by AIBN (Figure 7).

Table S3: *Experimental conditions for solution homopolymerization in toluene.*

Monomer	*MW* (g/mol)	*m* monomer (g)	*m* toluene (g)	*m* AIBN (g)	*m* IS (g)
**St**	104.15	0.81	2.7	0.01	0.090
**4VG**	150.15	1.19	3	0.017	0.090
**Ac4VG**	192.08	1.51	5.2	0.02	0.090
**Prop4VG**	206	1.61	5.4	0.025	0.080
**But4VG**	220.11	1.72	5.7	0.02	0.090
**Piv4VG**	234	1.81	5.8	0.024	0.090
**Hept4VG**	262.16	2.04	6.4	0.02	0.090
**Und4VG**	317.2	2.48	7.25	0.02	0.090
**HE4VG**	194	1.51	5.2	0.02	0.090
**AcHE4VG**	236.1	1.84	5.8	0.023	0.090
**Met4VG**	164.08	1.27	4.7	0.02	0.090

c) Experimental conditions and kinetics calculations for the ln(*[M]*$_0$/*[M]*) versus time plot for solution homopolymerization of styrene and 4VG-derivatives in deuterated toluene at 70°C initated by AIBN (Figure S23).

We considered:

ρ_{St} = 0.86 g/mL at 70°C [2]

$\rho_{d8\text{-}toluene}$ = 0.8932 g/mL at 70°C[3]

ρ_{IS} = 0.9 g/mL at 25°C[4]

A dry 50mL stainless steel paint density specific gravity liquid test cup (picnometer) was filled with 4VG$_{derivatives}$ (for a matter of quantity, only 4VG, Ac4VG and Hept4VG have been tested) from the top-hole. When the picnometer was fully filled, the fine hole started to release the monomer. At that moment, the addition was stopped, the mass measured (± 0.1 mg) and the density was calculated from the ***equation S9***, considering *V* = 50mL ± 0.05 mL.

$$\rho = \frac{m}{V} \qquad (\boldsymbol{equation\ S9})$$

The density measured by picnometry gave ρ_{4VG}=ρ_{Ac4VG}=$\rho_{Hept4VG}$=1.11 g/mL. Thus, in Table S4, as a first approximation, we have considered that ρ_{But4VG}=1.11 g/mL.

ρ_{AIBN} = 1.1 g/mL at 25°C[5]

Although the excess volume of mixing of the reactants is not expected to be null, it is usually small (*e.g.* 2.1% in the well-known case of ethanol-water mixture at 50mol% water[6]). We neglected this deviation to ideality as a first approximation and thus considered the additivity of the volumes of the reagents to calculate the concentration of *[AIBN]*$_0$ in Table S4.

$$ln\frac{[M]_{\mathbf{0}}}{[M]} = ln\frac{1}{1-r} = k_{\mathrm{p}} \times \sqrt{\frac{2fk_{\mathrm{d}}[I]}{gk_{\mathrm{t}}}} \times t \qquad (\boldsymbol{equation\ S10})$$

Where *[M]* is the concentration of monomer, *[I]* the initial concentration of initiator, *r* is the fractional conversion of monomer, k_p the propagation rate constant, k_t the termination rate constant (using R_t = -d*[P*]*/d*t* = *g*×k_t×*[P*]*^{2}, with *g*=2, i.e. IUPAC nomenclature), k_d =3.7 × 10^{-5} s^{-1} the decomposition rate constant of the initiator (AIBN)[7] and *f*=0.7 the initiator efficiency[8].

The concentration of initiator *[I]* (hence *[AIBN]*) was calculated from the ^{1}H-NMR spectra (mol) by using:

$$n_{\mathrm{AIBN,0}} = \frac{I_{\mathrm{AIBN,0}} \times n_{\mathrm{IS,0}}}{I_{\mathrm{IS,0}}} \qquad (\boldsymbol{equation\ S11})$$

The value of $k_p/(k_t^{0.5})$ can be calculated from the slope of Ln($[M]_0/[M]$)=f(t), where $slope = k_p/(2k_t)^{0.5}\times(2fk_d[AIBN]_0)^{0.5}$. Thus, $A = k_p/(k_t^{0.5}) = slope/(fk_d[AIBN]_0)^{0.5}$. Therefore, $\Delta A/A = (1/2)\times\Delta[AIBN]_0/[AIBN]_0 + \Delta slope/slope$. And $[AIBN]_0=n_{\mathrm{AIBN,0}}/V_{\mathrm{reaction\ mixture}}$, thus $\Delta[AIBN]_0/[AIBN]_0 = \Delta n_{\mathrm{AIBN,0}}/n_{\mathrm{AIBN,0}} + \Delta V/V$.

$n_{\mathrm{AIBN,0}}$ was calculated from the ^{1}H-NMR spectra at t_0: $n_{\mathrm{AIBN,0}} = (I_{\mathrm{AIBN,0}}\times n_{\mathrm{InternalStandard}})/I_{\mathrm{InternalStandard}}$, and $n_{\mathrm{InternalStandard}} = m_{\mathrm{InternalStandard}}/M_{\mathrm{InternalStandard}}$. Thus $\Delta n_{\mathrm{InternalStandard}}/n_{\mathrm{InternalStandard}} = \Delta m_{\mathrm{InternalStandard}}/m_{\mathrm{InternalStandard}}$ (uncertainty of balance $\Delta m_{\mathrm{InternalStandard}}$ = 0.1mg and $m_{\mathrm{InternalStandard}}$ = 40mg or 90mg, so $\Delta m_{\mathrm{InternalStandard}}/m_{\mathrm{InternalStandard}} < 0.25\%$). So, $\Delta n_{\mathrm{AIBN,0}}/n_{\mathrm{AIBN,0}} = 2\times\Delta I/I + \Delta n_{\mathrm{InternalStandard}}/n_{\mathrm{InternalStandard}} = 2\times1\% + 0.25\% = 2.25\%$ (where $\Delta I/I = 1\%$ by ^{1}H-NMR).

$\Delta V/V = 2\%$ (estimation of the volume uncertainty due to non-ideal mixture).

$\Delta[AIBN]_0/[AIBN]_0 = \Delta n_{\mathrm{AIBN,0}}/n_{\mathrm{AIBN,0}} + \Delta V/V = 2.25\% + 2\% = 4.25\%$.

Finally, it gives $\Delta A/A = \Delta[k_p/(k_t^{0.5})]/[k_p/(k_t^{0.5})] = (1/2)\times\Delta[AIBN]_0/[AIBN]_0 + \Delta slope/slope = (1/2)\times4.25\% + 2\% = 4.1\%$.

So the relative uncertainty of the estimated $k_p/(k_t^{0.5})$ values is 4.1%.

Table S4: *Experimental data for the radical homopolymerization of styrene and the 4VG derivatives in deuterated toluene at 70°C initiated by AIBN.*

Monomer	*MW* (g/mol)	*m* mon (g)	*V* mon (mL)	*m* toluene (g)	*V* toluene (mL)	*m* AIBN (g)	*V* AIBN (mL)	*m* IS (g)	*V* IS (mL)	*V* tot (mL)	*V* tot (L)	*n* AIBN (mol)	$[AIBN]_{t=0}$ (mol/L)	*slope*[a]	$k_p/(k_t^{0.5})$
**St**	104.15	0.8	0.93	2.7	3.02	0.0121	0.011	0.090	0.100	4.064	0.004	0.00007385	0.018	0.0000380	0.056
**Ac4VG**	192.08	0.76	0.68	2.6	2.91	0.0187	0.017	0.040	0.044	3.657	0.004	0.000113651	0.031	0.0001100	0.123
**But4VG**	220.11	1.72	1.55	5.7	6.38	0.0359	0.033	0.090	0.100	8.064	0.008	0.000218335	0.027	0.0000887	0.106
**Hept4VG**	262.16	2.04	1.84	6.4	7.17	0.0358	0.033	0.090	0.100	9.136	0.009	0.000218092	0.024	0.0001210	0.153

[a]*The slope values were calculated from **Figure S23**.*

d) Linear regressions of ln(1/(1-*r*)) versus time for **Figure S23**.

Table S5: *Experimental slopes and intercepts from the linear regressions of online measurement of* ***Figure S23***.

	Intercept		***Slope***		***Statistics***
	Value	***Standard Error***	***Value***	***Standard Error***	R^2
**St**	0.00438	0.00171	3.82E-05	7.95E-07	0.99183
**Ac4VG**	0.04547	0.00951	1.10E-04	3.11E-06	0.99683
**But4VG**	0.01020	0.00435	8.87E-05	1.97E-06	0.99461
**Hept4VG**	0.00786	0.00570	1.21E-04	2.58E-06	0.99500

e) Monomers integration and conversion at each time for homopolymerizations in deuterated toluene at 70°C initiated by AIBN, as reported in **Figure S23**.

Table S6: *Experimental data for monomer conversion at each time, reported in* ***Figure*** **S23**.

Integration IS (NMR)[a]	*t* (min)	*t* (s)	*Integration* St (NMR)	*Conversion* St (%)	ln(1/(1-*r*))	*t* (min)	*t* (s)	*Integration* Ac4VG (NMR)	*Conversion* Ac4VG (%)	ln (1/(1-*r*))	*t* (min)	*t* (s)	*Integration* But4VG (NMR)	*Conversion* But4VG (%)	ln (1/(1-*r*))	*t* (min)	*t* (s)	*Integration* Hept4VG (NMR)	*Conversion* Hept4VG (%)	ln (1/(1-*r*))
**100**	0	0	101.235	0	0	0	0	112.25	0.00	0.000	0	0	99.84	0.00	0.000	0	0	101.96	0	0.000
**100**	3	180	100	1.22	0.01	24	1440	83.07	17.94	0.198	5	300	97	4.18	0.043	5	300	99.64	0.36	0.004
**100**	6	360	99.11	2.10	0.02	36	2160	76.15	24.78	0.285	10	600	94.5	6.65	0.069	10	600	94.09	5.91	0.061
**100**	9	540	98.97	2.24	0.02	48	2880	69.8	31.05	0.372	15	900	90.71	10.40	0.110	15	900	90.34	9.66	0.102
**100**	12	720	98.46	2.74	0.03	60	3600	64.9	35.89	0.445	20	1200	88.2	12.88	0.138	20	1200	86.5	13.5	0.145
**100**	15	900	96.84	4.34	0.04						25	1500	86.53	14.53	0.157	25	1500	84.05	15.95	0.174
**100**	18	1080	96.41	4.77	0.05						30	1800	83	18.01	0.199	30	1800	81.31	18.69	0.207
**100**	21	1260	95.92	5.25	0.05						35	2100	82.1	18.90	0.210	35	2100	77.68	22.32	0.253
**100**	24	1440	96.22	4.95	0.05						40	2400	79.84	21.13	0.237	40	2400	75.38	24.62	0.283
**100**	27	1620	94.65	6.50	0.07						45	2700	78	22.95	0.261	45	2700	72.95	27.05	0.315
**100**	30	1800	93.95	7.20	0.07						50	3000	76.25	24.68	0.283	50	3000	70.83	29.17	0.345
**100**	33	1980	93.71	7.43	0.08						55	3300	73.75	27.15	0.317	55	3300	68.26	31.74	0.382
**100**	36	2160	92.98	8.15	0.09						60	3600	71.86	29.02	0.343	60	3600	65.95	34.05	0.416
**100**	39	2340	92.66	8.47	0.09															
**100**	42	2520	91.34	9.77	0.10															
**100**	45	2700	90.55	10.55	0.11															
**100**	48	2880	90.23	10.87	0.12															
**100**	51	3060	89.59	11.50	0.12															
**100**	54	3240	89.47	11.62	0.12															
**100**	57	3420	88.35	12.73	0.14															
**100**	60	3600	87.6	13.47	0.14															

[a]The integration of the IS had a value of 100.

Results of copolymerization

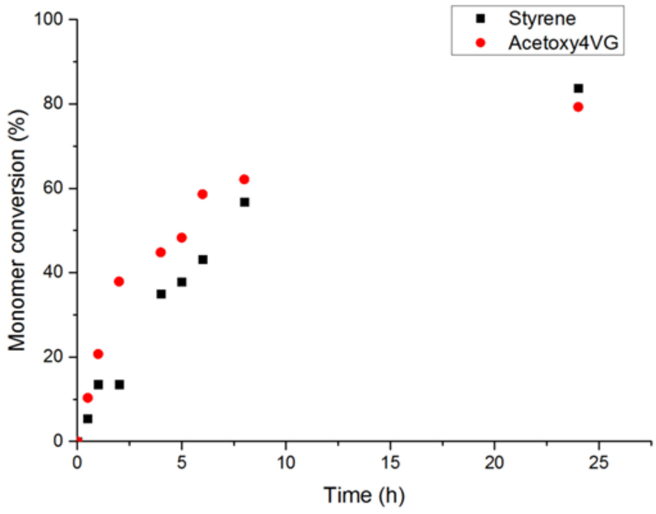

Figure S 24: *Monomer conversion in copolymerization of Ac4VG and Styrene in toluene solution initiated by AIBN at 70°C.*

Characterizations of the homopolymers

SEC results for homopolymers of 4VG derivatives:

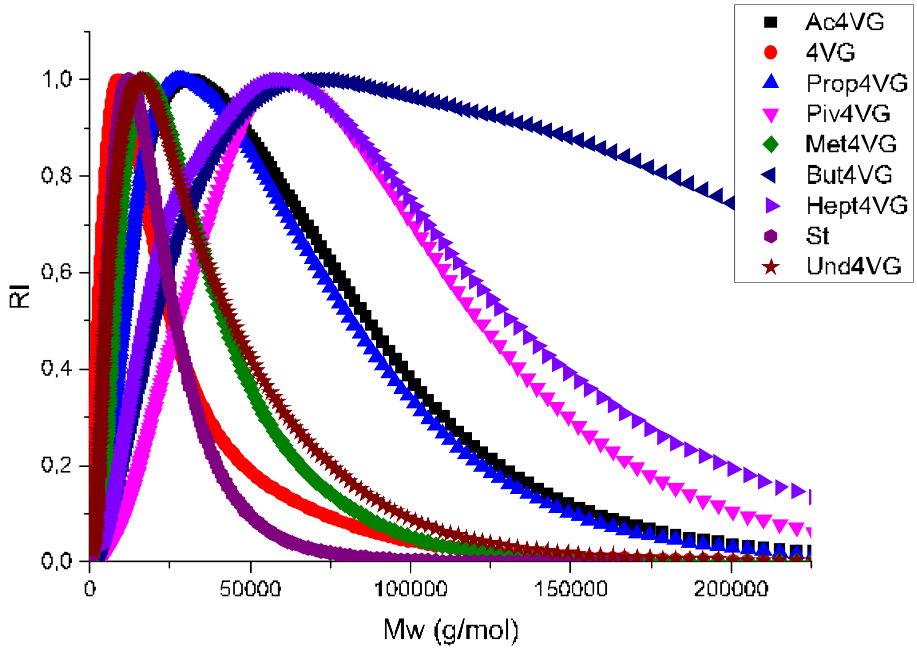

Figure S25: *SEC results of the produced homopolymers obtained by radical polymerization of the biobased 4VG derivatives initiated by AIBN at 70°C in toluene. SEC calibration was performed with PMMA narrow standards.*

TGA results for homopolymers of 4VG derivatives:

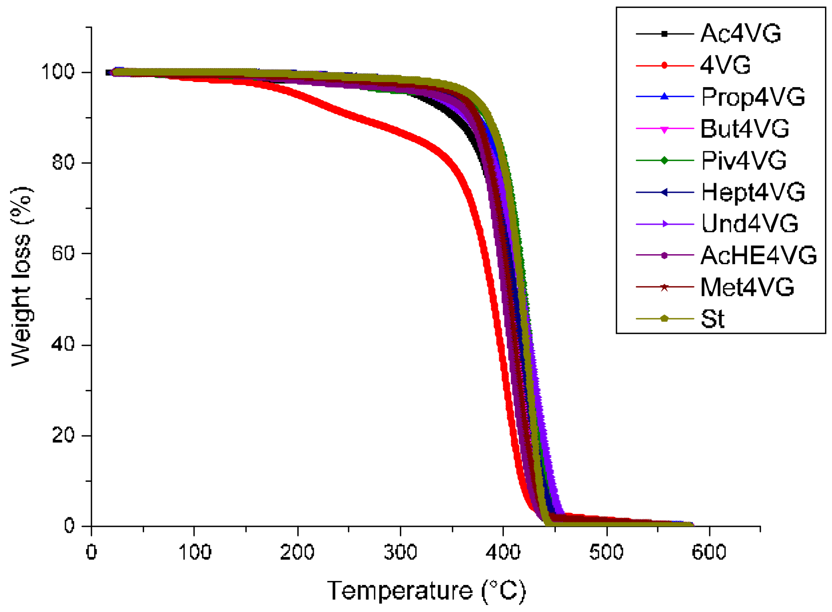

Figure S26: *Weight loss (%) versus temperature for the homopolymers (under nitrogen) obtained by solution radical polymerization of 4VG derivatives initiated by AIBN at 70°C in toluene.*

DSC results for homopolymers of 4VG derivatives:

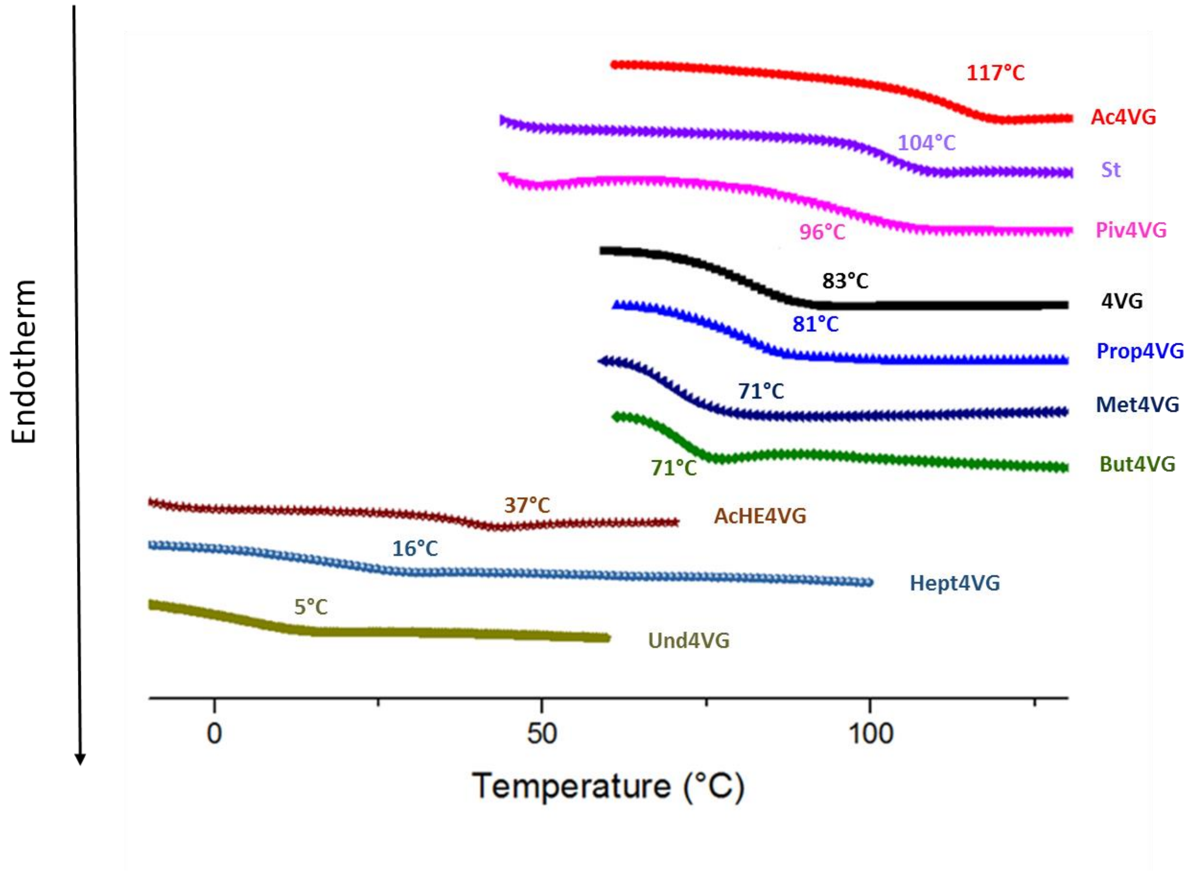

Figure S 27: *Differential scanning calorimetry (DSC) curves of the homopolymers obtained by solution radical polymerization of 4VG derivatives initiated by AIBN at 70°C in toluene.*

The glass transition temperatures of the polymers were calculated using the method of additive group contributions proposed by van Krevelen[9] using **equation S12**

$$T_g = \frac{\sum_i Y_i}{\sum_i M_i} \qquad \textbf{equation S12}$$

Table S7: *Group contributions corresponding to the chemical units.*

Group	Detail	Y_i (K kg mol^{-1})	M_i (g mol^{-1})
$—CH_2—$	In the main chain	2.7	14
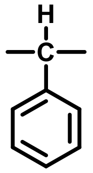		36.1	90.1
—CH(tert.butyl)		25.6	70.1
—O—		4	16
—C(=O)—	One side conjugated with aromatic ring	15.5	28
	Non-conjugated (isolated in aliphatic chain)	11.5	

Example of Styrene:

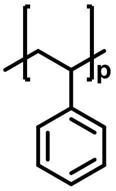

PStyrene

Figure S28 : *Chemical structure of polystyrene.*

Following ***equation S12*** and the data of ***Table S7,*** we obtained:

$$T_{\mathrm{g,PSt}} = \frac{1000 \ \times (2.7 + 36.1)}{14 + 90.1} = 373\ K = 100°C$$

Example of 4VG-derivatives:

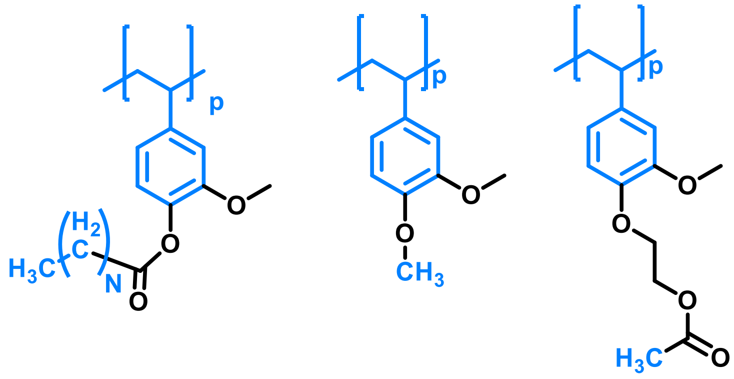

PAc4VG, PProp4VG, PBut4VG PHept4VG, PUnd4VG **PMet4VG** **PAcHE4VG**

Figure S29 : *Chemical structure of 4VG-derivatives.*

In our case, the homopolymers followed the series here below of polyalkylstyrene (poly(p-methylstyrene)) and the basic data to apply the formula were summarized in ***Table S8.***

Table S8: *Basic data for poly(p-methylstyrene) series.*

Structure of the polymer	Y_{g0} (K kg mol^{-1})	Y_{g9} (K kg mol^{-1})	M_0 (g mol^{-1})	M_9 (g mol^{-1})
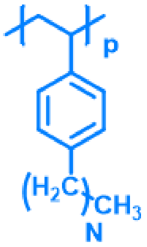	44.7	48.8	118.2	244.4

$$N < 9, \qquad Y_g \approx Y_{g0} + \frac{N}{9}\left(Y_{g9} - Y_{g0}\right) \qquad \textbf{equation } \boldsymbol{S13}$$

$$N = 9, \qquad Y_g \approx 0.2 M_9 = Y_{g9} \qquad \textbf{equation } \boldsymbol{S14}$$

From **Table S8 and equations S13 and S14** we obtained the following results:

Table S9: *Basic data for poly(p-methylstyrene) unit in 4VG-derivatives homopolymers.*

	N	**Y_g (K kg mol^{-1})**	***M* (g mol^{-1})**
**Ac4VG**	0	44.700	118.2
**Prop4VG**	1	45.156	132.2
**But4VG**	2	45.611	146.2
**Hept4VG**	5	46.978	188.3
**Und4VG**	9	48.880	244.4
**Met4VG**	0	44.700	118.2
**AcHE4VG**	2	45.611	146.2

From **Tables S7, S9** and **equation S12**, we were able to calculate the theoretical T_g of homopolymers as follows:

$$T_{\mathrm{g,PAc4VG}} = \frac{1000\ \times (44.7 + 4 \times 2 + 15.5)}{118.02 + 16 \times 2 + 28} = 383\ K = 110°C$$

$$T_{\mathrm{g,PProp4VG}} = \frac{1000\ \times (45.16 + 4 \times 2 + 15.5)}{132.2 + 16 \times 2 + 28} = 357\ K = 84°C$$

$$T_{\mathrm{g,PBut4VG}} = \frac{1000\ \times (45.6 + 4 \times 2 + 15.5)}{146.2 + 16 \times 2 + 28} = 335\ K = 62°C$$

$$T_{\mathrm{g,PHept4VG}} = \frac{1000\ \times (46.98 + 4 \times 2 + 15.5)}{188.3 + 16 \times 2 + 28} = 284\ K = 11°C$$

$$T_{\mathrm{g,PUnd4VG}} = \frac{1000\ \times (48.88 + 4 \times 2 + 15.5)}{244.4 + 16 \times 2 + 28} = 237\ K = -35°C$$

$$T_{\mathrm{g,PMet4VG}} = \frac{1000\ \times (44.7 + 4 \times 2)}{118.02 + 16 \times 2} = 351\ K = 78°C$$

$$T_{\mathrm{g,PAcHE4VG}} = \frac{1000\ \times (45.6 + 4 \times 3 + 11.5)}{146.2 + 16 \times 3 + 28} = 311\ K = 38°C$$

Example of Piv4VG:

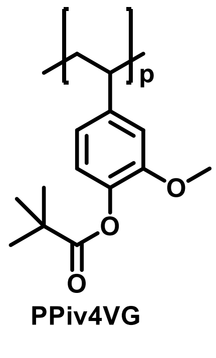

Figure S30 : *Chemical structure of polyPiv4VG.*

Following ***equation S12*** and the data of ***Table S7*** we obtained:

$$T_{\mathrm{g,PPiv4VG}} = \frac{1000\ \times (2.7 + 36.1 + 2\ \times 4 + 25.6 + 15.5)}{14 + 90.1 + 16 \times 2 + 70.1 + 28} = 275\ K = 102°C$$

The results can be summarized as follows:

Table S10 : *Comparison between T_g values of Styrene and 4VG-derivatives calculated by van Krevelen method and measured by DSC.*

	$T_{g,calculated}$ (°C)	$T_{g,measured}$ by DSC (°C)
**St**	100	104
**Ac4VG**	110	117
**Prop4VG**	84	81
**But4VG**	62	71
**Piv4VG**	102	96
**Hept4VG**	11	16
**Und4VG**	-35	5
**Met4VG**	78	71
**AcHE4VG**	38	37

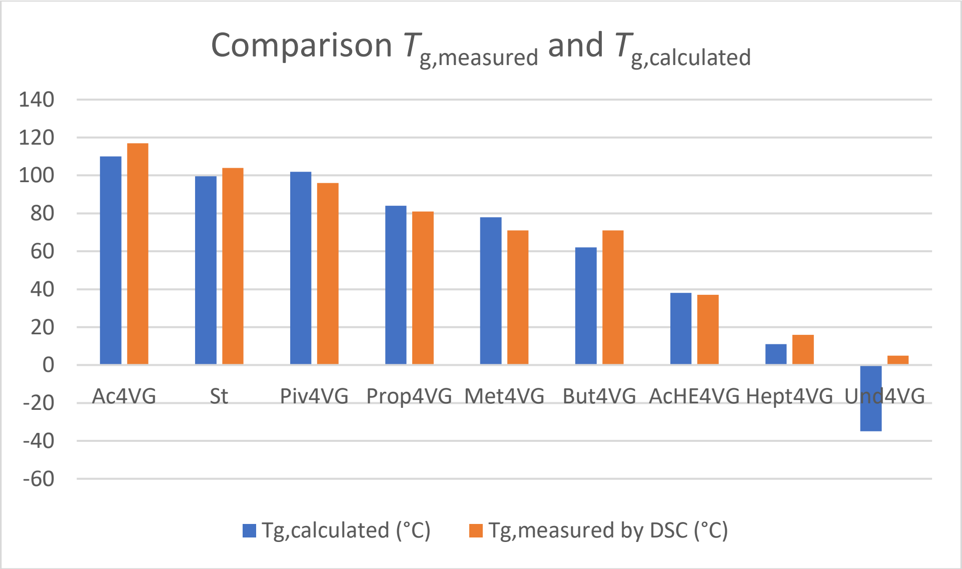

Figure S31 : *Graphical representation of T_g comparison summarized in **Table S10**.*

References

1. Bauer, M.; Bertario, A.; Boccardi, G.; Fontaine, X.; Rao, R.; Verrier, D. Reproducibility of 1H-NMR Integrals: A Collaborative Study. *J. Pharm. Biomed. Anal.* **1998**, *17*, 419–425, doi:10.1016/S0731-7085(97)00208-2.

2. Patnode, W.; Scheiber, W.J. The Density, Thermal Expansion, Vapor Pressure, and Refractive Index of Styrene, and the Density and Thermal Expansion of Polystyrene. *J. Am. Chem. Soc.* **1939**, *61*, 3449–3451, doi:10.1021/ja01267a066.

3. Killian, W.G.; Norfleet, A.T.; Lira, C.T. Densities of Selected Deuterated Solvents. *J. Chem. Eng. Data* **2022**, *67*, 893–901, doi:10.1021/acs.jced.1c00990.

4. www.chemspider.com/Chemical-Structure.24010.html 1,4-Bis(Trimethylsilyl)Benzene. accessed on 16 April 2024.

5. Roduit, B.; Hartmann, M.; Folly, P.; Sarbach, A.; Brodard, P.; Baltensperger, R. Determination of Thermal Hazard from DSC Measurements. Investigation of Self-Accelerating Decomposition Temperature (SADT) of AIBN. *J. Therm. Anal. Calorim.* **2014**, *117*, 1017–1026, doi:10.1007/s10973-014-3903-3.

6. Franks, F.; Ives, D.J.G. The Structural Properties of Alcohol–Water Mixtures. *Q. Rev. Chem. Soc.* **1966**, *20*, 1–44, doi:10.1039/QR9662000001.

7. J. Van Hook J. Tobolsky The Thermal Decomposition of 2-2-Azo-Bis-Isobutyronitrile. *J. Am. Chem. Soc.* **1958**, *80*, 779–782.

8. Lacroix-Desmazes, P.; Severac, R.; Boutevin, B. Reverse Iodine Transfer Polymerization of Methyl Acrylate and N-Butyl Acrylate. *Macromolecules* 2005, *38*, 6299–6309.

9. Van Krevelen, D.W. *Properties of Polymers: Their Correlation with Chemical Structure; Their Numerical Estimation and Prediction from Additive Group Contributions*; Elsevier, Ed.; Fourth edi.; 2009; ISBN 9780080548197.
